# Diabetes-related exposure and screening-derived abnormality burden among older rural women in Northeast China: a secondary analysis with contextual labour-type physical activity assessment

**DOI:** 10.3389/fpubh.2026.1841622

**Published:** 2026-07-01

**Authors:** Limeng Liu, Yongheng Zhao, Xi Xuefeng, Gaixia Hou, Yanqiu Liu, Yong Wang, Dehui Zhang

**Affiliations:** 1 College of Physical Education and Health Sciences, Mudanjiang Normal University, Mudanjiang, China; 2 School of Wushu, Henan University, Kaifeng, China; 3 School of Physical Education, Sanming University, Sanming, China; 4 Dongcheng Community Health Service Center, Wangkui, China

**Keywords:** community health examination, contextual labour-type physical activity, diabetes-related exposure, healthy aging, integrated care, older women, rural primary care, screening-derived abnormality burden

## Abstract

**Background:**

Routine primary-care health examinations can identify clustered metabolic, renal–urinary, medication-related, and functional screening abnormalities in older adults, but their value for screening-derived abnormality-burden assessment in rural ageing populations remains underexplored.

**Methods:**

This community-based cross-sectional secondary analysis used 2025 annual health-examination data from Wangkui County, Heilongjiang Province, Northeast China. The analytic sample included 1,163 women aged ≥65 years. High screening-derived abnormality burden was constructed from routinely available screening indicators and was not intended to diagnose constipation, bowel dysfunction, frailty, or any clinical disease endpoint. Diabetes-related exposure was defined as self-reported diabetes, fasting plasma glucose ≥7.0 mmol/L, or glucose-lowering medication use. Labour-type physical activity was assessed as a secondary contextual routine-record variable using a cohort-specific index derived from weekly frequency and duration. Associations were estimated using modified Poisson regression with robust HC3 standard errors.

**Results:**

High screening-derived abnormality burden was present in 27.52% of participants, and diabetes-related exposure was present in 22.53%. Diabetes-related exposure was associated with higher abnormality burden in the primary adjusted model and remained similar after BMI adjustment (PR 1.614, 95% CI 1.329–1.961). In the key non-metabolic sensitivity analysis excluding triglycerides, HDL-C, and BMI abnormality, diabetes-related exposure remained positively associated with high non-metabolic abnormality burden (PR 1.463, 95% CI 1.077–1.988). LowActive and HighActive were not independently associated with high abnormality burden and were interpreted as contextual findings rather than as primary physical-activity evidence.

**Conclusion:**

Among older rural women, diabetes-related exposure was associated with higher screening-derived abnormality burden. Labour-type physical activity did not independently distinguish abnormality-burden status under the available crude routine-record measurement framework. This internally derived outcome should be interpreted as exploratory clustering of routine screening abnormalities for primary-care triage, not as a validated geriatric phenotype or clinical diagnostic endpoint. From an aging-and-public-health perspective, these findings suggest that routine older-adult health examinations in underserved rural communities may provide a scalable opportunity for population-level risk recognition, follow-up prioritisation, health guidance, and referral review among older women living with diabetes-related screening burden, while avoiding diagnostic over interpretation.

## 1 Introduction

Population ageing has substantially increased the burden of chronic disease, multimorbidity, and functional vulnerability among community-dwelling older adults worldwide (1, 2). In routine primary-care settings, these burdens are often observed not as isolated conditions, but as clusters of metabolic, renal–urinary, medication-related, and functional screening abnormalities during annual health examinations (3, 4). Older women may show a high burden of such clustering because ageing is frequently accompanied by changes in body composition, chronic disease accumulation, treatment exposure, and declining physiological reserve (5, 6). However, screening-derived abnormality burden among older rural women remains insufficiently characterised, especially in underrepresented cold-climate agricultural regions (7).

Routine community health-examination platforms provide an opportunity to recognise clustered screening abnormalities in older adults, but they are not designed to replace symptom-oriented clinical assessment. In particular, annual primary-care examinations often do not include structured bowel-symptom instruments, stool diaries, Bristol stool form assessment, stool-frequency records, Rome criteria, or clinician-adjudicated constipation diagnoses (8, 9). Therefore, such data are not suitable for estimating constipation prevalence or diagnosing symptom-defined bowel disease. The present study should instead be understood as an exploratory primary-care screening analysis of routinely recorded abnormality burden. Constipation and bowel symptoms are clinically relevant concerns in older adults, particularly among older women, but symptom prevalence estimates depend strongly on diagnostic criteria and data-collection instruments. In rural primary-care screening programmes, such symptom-specific information is often unavailable, which limits direct estimation of constipation prevalence but creates a practical need for transparent screening-oriented abnormality-burden measures.

In symptom-unavailable screening settings, a screening-derived abnormality-burden outcome may provide a pragmatic approach for identifying older adults with clustered routine abnormalities who may warrant further clinical review. This internally derived outcome should not be interpreted as a validated multidomain geriatric vulnerability phenotype, a surrogate diagnosis of constipation, bowel dysfunction, a frailty index, or a comprehensive geriatric assessment score. Rather, it summarises routinely recorded metabolic, renal–urinary, medication-related, and functional abnormalities that may reflect broader screening burden and the need for more detailed assessment (10, 11). Its value lies in risk recognition, triage, and community-level prioritisation, not in replacing symptom-based bowel assessment, comprehensive geriatric evaluation, or clinician-adjudicated diagnosis. Framed as an aging-and-public-health question, the present study asks whether routine health-examination data can support scalable and equity-oriented risk recognition among older women in an underserved rural, cold-climate setting. This framing links diabetes-related screening burden with healthy-aging support, chronic-disease management, functional and medication-related review, and integrated primary-care/public-health follow-up within an existing community health-examination system.

Diabetes-related exposure may be especially relevant within this screening framework. In later life, glycaemic screening burden often coexists with dyslipidaemia, body-composition abnormality, renal–urinary abnormalities, treatment complexity, and functional limitation (12, 13). Because the proposed abnormality-burden outcome includes metabolic components, conceptual overlap between diabetes-related exposure and outcome components must be interpreted cautiously. Although diabetes and hyperglycaemia have been linked to gastrointestinal symptoms and autonomic complications in disease-specific research, these pathways cannot be inferred without symptom-based or complication-specific measurements (14, 15). In routine community health-examination systems, diabetes-related exposure is therefore better interpreted as a pragmatic marker of clustered metabolic and systemic screening burden rather than as evidence of a specific bowel-disease process.

Physical activity is another important but context-dependent factor in later-life health research (16, 17). Many studies associate higher physical activity with healthier ageing and lower chronic disease burden, but these findings often rely on leisure-time exercise, guideline-based activity measures, validated physical-activity questionnaires, or objective monitoring (18, 19). In rural agricultural settings, especially in cold-climate regions, recorded activity may reflect labour demand, domestic responsibility, seasonal work, and survival-oriented movement rather than structured health-promoting exercise (20, 21). Accordingly, labour-type physical activity among older rural women may represent work obligation and household responsibility rather than restorative or leisure-time exercise. Because the available activity measure was cohort-specific and based only on routine records of frequency and duration, measurement imprecision is possible, and any null association should be interpreted cautiously.

This issue is particularly relevant in rural Northeast China, where older adults live in a context shaped by agricultural work patterns, seasonal environmental constraints, dietary and lifestyle characteristics, and community-based primary-care screening structures (3, 4, 7). In such settings, annual health-examination data may be better suited to identifying clusters of metabolic, renal–urinary, medication-related, and functional screening abnormalities than to identifying symptom-defined constipation directly. This provides an opportunity to examine whether routinely available screening indicators can characterise screening-derived abnormality burden among older rural women and whether diabetes-related exposure identifies women with a higher burden of clustered routine screening abnormalities.

Against this background, the present study focused on older women participating in a rural community health-examination programme in Northeast China. We did not attempt to diagnose constipation, estimate constipation prevalence, or define symptom-based bowel disease. Instead, we examined an exploratory screening-derived abnormality-burden outcome based on routinely available primary-care indicators. The primary aim was to characterise the distribution of high screening-derived abnormality burden and to examine its association with diabetes-related exposure among older rural women. Labour-type physical activity was retained as a secondary contextual routine-record variable because the available measure reflected crude frequency and duration of labour-related activity rather than validated exercise dose, leisure-time physical activity, or objectively measured energy expenditure. Given the potential overlap between diabetes-related exposure and metabolic outcome components, we also planned a non-metabolic sensitivity analysis to assess whether the observed diabetes-related association was fully explained by shared metabolic abnormalities. By using routinely collected data from the older-adult health-examination platform under the National Basic Public Health Service framework, this study may inform risk triage and follow-up prioritisation in community-based primary care rather than disease diagnosis.

The present manuscript is related to two previous publications by overlapping members of the author group that used the same parent Wangkui County routine health-examination database (7, 22). Those previous studies focused, respectively, on labour-type physical activity, alcohol use, and hypertension in the overall rural older-adult population, and on occupational/labour-type physical activity, total diabetes burden, the triglyceride–glucose index, and urine occult blood positivity in the overall rural older-adult population. The current study differs by restricting the analytic population to older rural women and by using cumulative screening-derived abnormality burden as the primary outcome. Accordingly, the present analysis addresses a distinct female-specific primary-care screening question rather than repeating the primary hypotheses or primary endpoints of the previous publications.

## 2 Methods

### 2.1 Study design, data source, and ethics

This study was designed as a community-based cross-sectional analysis using routinely collected annual health-examination data from older adults in a rural area of Northeast China. The data were derived from the National Basic Public Health Service older adult health-examination platform, which provides annual health management for residents aged 65 years and older, including lifestyle and health-status assessment, physical examination, auxiliary examination, and health guidance (3, 4). The use of the 2025 older adult health-examination data was authorised by Wangkui County Dongcheng Community Health Service Center. According to the data authorisation letter, the dataset included routine public-health examination information for residents aged 65 years or older, including demographic characteristics, chronic-disease management indicators, anthropometric measurements, vital signs, laboratory tests, health behaviours, and follow-up records. The data provider stated that personal identifiers, including names and identity information, had been removed before data transfer. The dataset was used only for scientific research and analysis, and all data handling followed the agreed requirements for de-identification, minimum necessary use, information security, and restricted access.

The 2025 Wangkui County older adult health-examination source platform has supported previous analyses by overlapping members of the author group. The relationship between the present manuscript and those previous publications is described explicitly below. The present study focuses on older rural women to reduce sex-related heterogeneity and improve interpretability within this screening-based framework.

**Figure 1:**
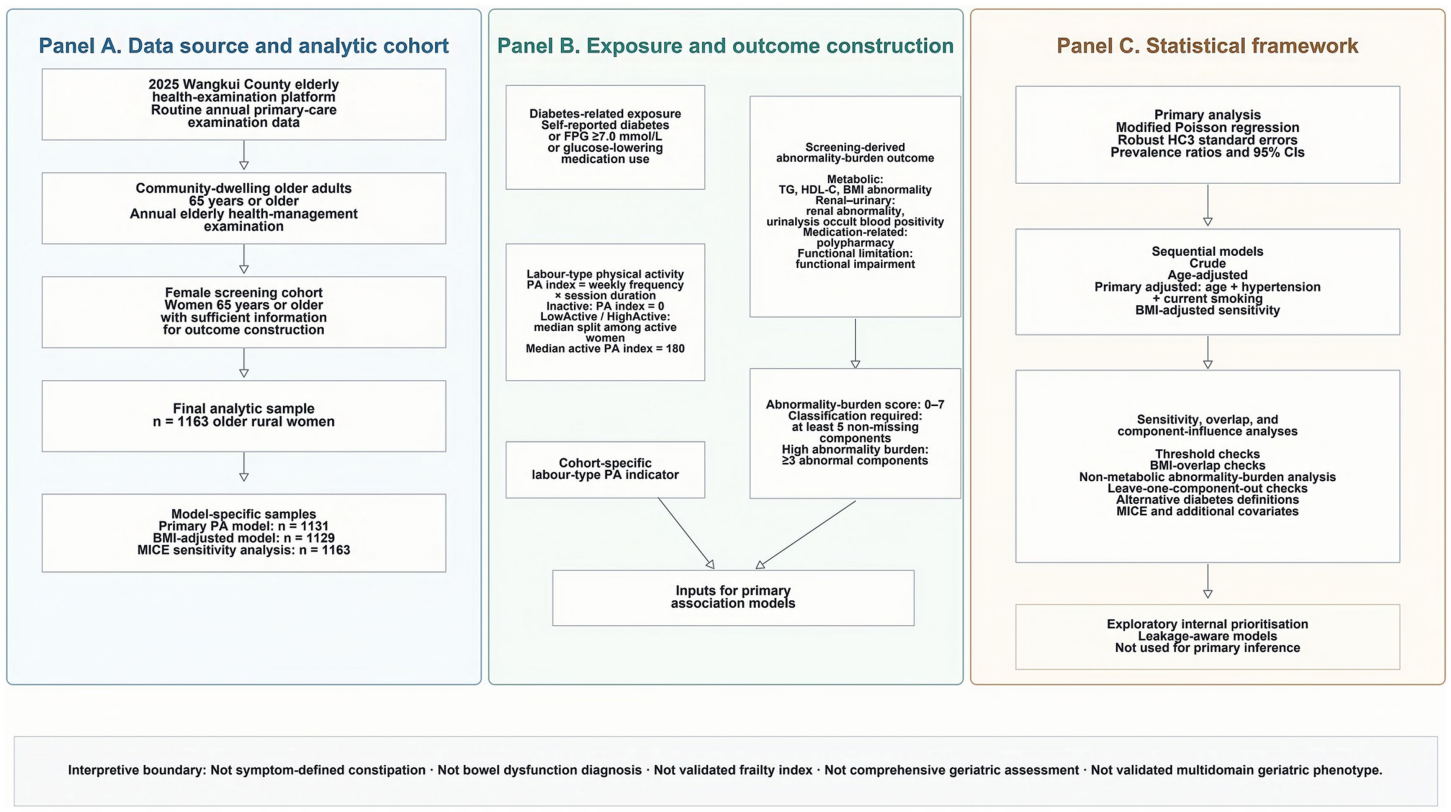
Study flowchart and analytic framework for the female screening cohort.

The annual examination programme included demographic information, lifestyle behaviours, physical examination findings, laboratory indicators, medication-related information, and selected functional assessments. The platform was established for routine primary-care screening rather than symptom-oriented gastrointestinal evaluation. It did not include Rome IV criteria, stool diaries, Bristol stool form assessment, stool-frequency records, or clinician-adjudicated constipation diagnosis (8, 9). Accordingly, the present study did not aim to diagnose constipation, estimate constipation prevalence, or define bowel dysfunction in the conventional clinical sense. Instead, it examined an internally derived screening-derived abnormality-burden outcome based on routinely available primary-care screening indicators. The overall study workflow, including participant selection, exposure assessment, outcome construction, missing-data handling, and statistical analysis, is shown in Figure 1.

This study was conducted in accordance with the 2024 revision of the Declaration of Helsinki (23). The study protocol was approved by the Biomedical Research Ethics Committee of Henan University, with the approval number HUSOM2025-929 and approval date of 12 November 2025. The present analysis used de-identified secondary health-examination data obtained through institutional data authorisation. Individual informed consent for this secondary analysis was handled according to the approved ethics protocol and local health-data governance requirements.

### 2.2 Relationship with previous publications

The present manuscript was conducted as a secondary analysis using the same parent routine health-examination database from Wangkui County, Northeast China, that supported two previous publications by overlapping members of the author group. One previous publication examined labour-type physical activity and alcohol use in relation to measured hypertension in the overall rural older-adult population. Another previous publication examined occupational/labour-type physical activity in relation to total diabetes burden, the triglyceride–glucose index, and urine occult blood positivity in the overall rural older-adult population.

The current study differs from these previous analyses in its analytic population, primary outcome structure, and public-health interpretation. First, the analytic population was restricted to older rural women rather than the overall older-adult population. Second, the primary outcome was a cumulative screening-derived abnormality-burden measure constructed from routinely available primary-care examination indicators, rather than hypertension, total diabetes burden, the triglyceride–glucose index, or urine occult blood positivity as a standalone endpoint. Third, the main research question was whether diabetes-related exposure identifies older rural women with clustered routine screening abnormalities who may warrant follow-up prioritisation in primary care. Labour-type physical activity was retained as a secondary contextual routine-record variable and was not the primary conceptual contribution of the present study.

Accordingly, although the source database and some participants overlap with previous publications, the present manuscript addresses a female-specific primary-care screening question, uses a restricted analytic sample, applies a different primary outcome structure, and provides a different interpretation from the previous publications.

### 2.3 Study population

Participants were eligible if they were women aged 65 years or older who completed the annual community health examination during the study period and had sufficient information for construction of the screening-derived abnormality-burden outcome. Women were excluded if key information on the primary exposure, outcome components, or core analytical variables was entirely unavailable.

For outcome construction, participants were required to have at least five non-missing component indicators, so that classification would not rely on excessively sparse information. This rule was applied to improve the stability and interpretability of the screening-derived abnormality-burden classification. The final female analytic sample included 1,163 older women. Because some regression models required complete information on labour-type physical-activity classification or selected covariates, the effective sample size varied slightly across models and was reported for each analysis.

### 2.4 Diabetes-related exposure

The primary exposure was diabetes-related exposure, defined to capture glycaemic screening burden within the routine health-examination setting. Participants were classified as having diabetes-related exposure if they met at least one of the following conditions: self-reported history of diabetes, recorded use of glucose-lowering medication, or fasting plasma glucose ≥7.0 mmol/L, consistent with the fasting-glucose diagnostic threshold specified in the American Diabetes Association Standards of Care in Diabetes—2026 (12).

Because the health-examination platform did not include repeated confirmatory testing, diabetes duration, glycaemic history, complication adjudication, or treatment-intensity information, this variable was interpreted as diabetes-related exposure or glycaemic screening burden rather than as a complete clinical characterisation of diabetes diagnosis, severity, or complications. To evaluate whether the findings depended on the medication component of the exposure definition, sensitivity analyses repeated the primary models using three alternative definitions: self-reported diabetes or fasting plasma glucose ≥7.0 mmol/L without glucose-lowering medication use, fasting plasma glucose ≥7.0 mmol/L only, and self-reported diabetes only.

### 2.5 Secondary contextual labour-type physical activity assessment

Labour-type physical activity was assessed as a secondary contextual routine-record variable rather than as the primary conceptual exposure of the present study. It was retained to describe the rural screening context, to account for crude labour-related activity information available in the routine examination platform, and to support transparent comparison with previous analyses from the same parent database. Physical activity was assessed using routine examination questions on weekly activity frequency and session duration, with broad activity descriptions including walking, agricultural labour, and domestic work. The examination platform did not collect standardised physical-activity questionnaire data, objectively measured activity, domain-specific intensity, leisure-time exercise dose, occupational workload, or total energy expenditure. Therefore, this variable was interpreted as a crude, cohort-specific, routine-record-based labour-type physical-activity indicator rather than as a validated exercise-dose measure, leisure-time physical-activity measure, guideline-based physical-activity category, or standard health-promoting exercise indicator (16, 18).

A cohort-specific physical-activity index was calculated as weekly activity frequency × session duration. Frequency responses were harmonised into weekly frequency values. Responses indicating no activity were assigned a frequency of 0, occasional activity was coded as 2 times per week, and frequent or regular activity was coded as 7 times per week; numeric frequency values, when available, were retained as recorded. For participants reporting no activity, missing duration was treated as 0. Participants with a physical-activity index of 0 were classified as Inactive. Among active women, the median physical-activity index was 180; values below this median were classified as LowActive, and values at or above this median were classified as HighActive. These categories represented relative labour-type activity levels within this cohort and were not intended to correspond to international guideline-based physical-activity categories, validated exercise-dose levels, or objectively measured activity intensity.

The labour-type physical-activity variable was represented as a three-level contextual variable: Inactive, LowActive, and HighActive. Because the LowActive stratum was comparatively modest in size, LowActive and HighActive were additionally collapsed into a binary Active category for robustness and descriptive analyses. Estimates involving labour-type physical activity were interpreted as secondary contextual findings rather than as primary evidence regarding the health effects of occupational or leisure-time physical activity.

### 2.6 Screening-derived abnormality burden outcome

Because the annual health-examination platform did not include symptom-based bowel assessment tools or validated geriatric vulnerability instruments, the present study did not attempt to define constipation, bowel dysfunction, frailty, or a clinical geriatric syndrome. Instead, we constructed an internally derived exploratory screening-derived abnormality burden outcome to summarise the clustering of routinely observable metabolic, renal–urinary, medication-related, and functional screening abnormalities in older women. This outcome was designed for primary-care screening interpretation only and should not be regarded as a clinical diagnosis, a validated multidomain geriatric vulnerability phenotype, a validated frailty measure, a comprehensive geriatric assessment score, or a disease-specific endpoint.

The outcome comprised seven routinely recorded screening abnormalities across four organisational domains: metabolic burden, renal–urinary burden, medication-related burden, and functional limitation. These domains were used to organise routinely available indicators only and were not intended to imply a balanced, weighted, or externally validated multidomain geriatric phenotype. All components were equally coded, and no psychometric testing, formal weighting, or empirical calibration was performed. The metabolic components were elevated triglycerides, operationalised as triglycerides ≥1.7 mmol/L; low HDL-C, operationalised as HDL-C < 1.3 mmol/L; and abnormal BMI, operationalised as BMI < 18.5 kg/m^2^ or BMI ≥ 28 kg/m^2^, with the obesity cut-off aligned with Chinese adult BMI classification (24). The renal–urinary components were renal abnormality, operationalised as creatinine >84 μmol/L and/or urea >7.1 mmol/L according to the routine laboratory coding used in the examination dataset, and urinalysis occult blood positivity, operationalised as trace or greater occult-blood positivity, positive coding, or equivalent positive coding on routine urinalysis. The medication-related component was polypharmacy, operationalised as an existing polypharmacy flag in the routine record or the concurrent use of five or more medications when medication-count information was available (25). The functional component was functional impairment, operationalised as ADL impairment and/or recorded disability in the annual health-examination platform.

Each component was coded as 1 if present and 0 if absent, yielding a total score ranging from 0 to 7. Participants were required to have at least five non-missing component indicators for classification. The primary operational definition of high screening-derived abnormality burden was the presence of three or more abnormal components. This threshold was selected to identify participants with co-occurring routine screening abnormalities rather than isolated single-component abnormality, while retaining sufficient prevalence and model stability for regression analyses. Because this score was internally derived, unweighted, and not externally validated or empirically calibrated, it was interpreted as an exploratory screening-burden measure rather than as a validated clinical, prognostic, or geriatric phenotype. Alternative thresholds of two or more and four or more abnormal components were examined in sensitivity analyses.

This outcome should not be interpreted as a surrogate diagnosis of constipation, a measure of constipation prevalence, a symptom-defined bowel-dysfunction endpoint, a validated frailty index, a comprehensive geriatric assessment score, or a disease-specific clinical endpoint. Rather, it should be understood as a pragmatic screening-derived abnormality-burden outcome reflecting the co-occurrence of routinely observable metabolic, renal–urinary, medication-related, and functional screening abnormalities in older women. Because the components were not equally prevalent and the score was shaped by routinely available examination indicators, the outcome should not be interpreted as a balanced multidomain geriatric vulnerability phenotype. None of the components was treated as bowel-specific or diagnostic of constipation.

The conceptual rationale and interpretive boundary for each component were summarised in Supplementary Table S1. The prevalence of each abnormality-burden component is summarised in Supplementary Table S3.

### 2.7 Covariates

Covariates were selected before fitting the primary models on the basis of clinical plausibility, epidemiological relevance, and availability within the routine screening platform. Age, hypertension, and current smoking were included in the primary adjusted model.

Continuous BMI was not included in the primary adjusted model because abnormal BMI was one component of the screening-derived abnormality-burden outcome. Including BMI in the primary model could therefore introduce overlap between an abnormality-burden component and a model covariate, potentially complicating interpretation of the exposure–outcome association. Instead, BMI adjustment was evaluated in a separate sensitivity model to assess the robustness of the primary findings. An additional sensitivity analysis was also conducted after excluding the BMI-abnormality component from the abnormality-burden score.

Covariates were incorporated into multivariable models to reduce confounding while preserving a parsimonious and interpretable cross-sectional framework. Because the routine examination platform did not include detailed information on diet quality, hydration, bowel symptoms, depressive symptoms, medication classes beyond the available medication records, objectively measured physical activity, or validated leisure-time physical-activity assessment, residual confounding and measurement-related uncertainty could not be fully eliminated.

### 2.8 Primary statistical analysis

All analyses followed a cross-sectional analytical framework. Continuous variables were summarised as means with standard deviations or medians with interquartile ranges, as appropriate, and categorical variables were summarised as counts and percentages. For descriptive baseline comparisons, Student’s *t*-test was used for continuous variables, and chi-square tests or Fisher’s exact tests were used for categorical variables, as appropriate. These *p* values were descriptive only and were not used for variable selection. Because several baseline variables were components or component-related measures of the screening-derived abnormality-burden outcome, between-group comparisons were not interpreted as independent explanatory or etiological factors for high abnormality burden.

The primary analysis examined the association between diabetes-related exposure and high screening-derived abnormality burden using modified Poisson regression with robust HC3 standard errors. The three-level labour-type physical-activity variable was included as a secondary contextual routine-record variable and was interpreted separately from the primary diabetes-related association. This approach was selected because the outcome was binary and relatively common, and prevalence ratios are more directly interpretable than odds ratios in cross-sectional screening data (26). Robust HC3 standard errors were used to reduce sensitivity to heteroscedasticity and influential observations in this screening dataset.

Three models were fitted sequentially. Model 1 was unadjusted. Model 2 adjusted for age. Model 3 was the primary adjusted model and included age, hypertension, and current smoking. Continuous BMI was not included in Model 3 because abnormal BMI was part of the screening-derived abnormality-burden outcome. A BMI-adjusted model was fitted as a sensitivity analysis to examine whether the primary findings were robust to additional adjustment for continuous BMI despite potential overlap with an outcome component.

Because the three-level physical-activity framework was cohort-specific and the low active stratum was comparatively modest in size, a robustness analysis was performed using the binary Active versus Inactive specification. A descriptive stratified analysis according to diabetes-related exposure and binary labour-type physical activity was also conducted for transparency. This analysis was descriptive, was not used to support the primary inference, and was not intended to estimate causal interaction or to advance a primary physical-activity hypothesis.

### 2.9 Sensitivity analyses and missing-data handling

Several sensitivity analyses were conducted to evaluate threshold dependence, alternative outcome specifications, and component influence, potential overlap between exposure definitions and outcome components, and robustness to missing-data handling.

First, alternative thresholds of two or more and four or more abnormal components were applied to the abnormality-burden score. Second, the screening-derived abnormality-burden outcome was analysed as an ordered categorical outcome and as a count-based score, rather than solely as a binary high abnormality-burden outcome. Third, BMI overlap was assessed by repeating the primary model with additional BMI adjustment and by recalculating the abnormality-burden outcome after excluding the BMI-abnormality component. Fourth, a non-metabolic abnormality-burden outcome was constructed after excluding triglycerides, HDL-C, and BMI abnormality. This non-metabolic outcome included renal abnormality, urinalysis occult blood positivity, polypharmacy, and functional impairment, with high non-metabolic abnormality burden defined as two or more abnormal non-metabolic components. This analysis was treated as a key interpretive sensitivity analysis because the primary outcome included metabolic components that could conceptually overlap with diabetes-related exposure.

Fifth, domain-specific analyses were conducted for metabolic burden, renal–urinary burden, and medication–functional burden. Metabolic burden was defined as two or more abnormalities among triglycerides, HDL-C, and BMI abnormality. Renal–urinary burden was defined as renal abnormality and/or urinalysis occult blood positivity. Medication–functional burden was defined as polypharmacy and/or functional impairment. These domain-specific analyses were exploratory because the number and prevalence of components differed across domains; they were used to examine component contribution and outcome consistency rather than to support domain-specific mechanistic conclusions.

Sixth, leave-one-component-out analyses were performed by removing each of the seven abnormality-burden components one at a time and recalculating high screening-derived abnormality burden using the remaining components. Seventh, alternative diabetes-related exposure definitions were examined to assess whether the findings depended on glucose-lowering medication use, laboratory-defined glycaemic burden, or self-reported diabetes. Additional covariate-adjusted sensitivity analyses further included routinely available screening covariates when applicable. Key sensitivity analyses addressing BMI overlap, metabolic-component dominance, non-metabolic abnormality burden, alternative diabetes definitions, missing-data handling, and additional covariate adjustment were summarised in the main text; full model-specific results were reported in the Supplementary Material.

These analyses were intended to examine the stability and interpretability of the observed pattern rather than to redefine the primary outcome or validate the internally derived score as a clinical, prognostic, geriatric, or bowel-specific endpoint. Sensitivity analyses were interpreted according to direction, magnitude, confidence intervals, and consistency across specifications rather than isolated statistical significance. No formal multiplicity correction was applied because these analyses were designed to examine robustness, component influence, and outcome specification rather than to test independent confirmatory hypotheses.

Missing-data handling was purpose-specific. For outcome construction, participants were required to have at least five non-missing component indicators to be classified as having high screening-derived abnormality burden or not, thereby avoiding unstable classification based on overly sparse component information. For the primary regression analyses, complete-case modelling was used to preserve consistency across the model covariate set; consequently, the effective sample size varied slightly across models.

Missingness was summarised for all primary exposures, outcome components, and model covariates. To evaluate potential selection related to complete-case modelling, participants included in and excluded from the primary adjusted model were compared descriptively.

To assess whether the main inference was materially altered by missing-data handling, multiple-imputation analyses using chained equations were conducted (27). Twenty imputed datasets were generated, with 10 iterations per imputation. The imputation model included the outcome as a predictor but did not impute the outcome itself, and also included diabetes-related exposure, labour-type physical activity, and model covariates used in the BMI-adjusted primary sensitivity model. Binary variables were imputed using logistic regression, multi-category variables using polytomous regression, and continuous variables using predictive mean matching. Modified Poisson models with robust HC3 standard errors were fitted within each imputed dataset, and estimates were pooled using Rubin’s rules (28).

### 2.10 Supplementary exploratory prediction analysis, software, and reporting

A supplementary leakage-aware internal prediction analysis was conducted only for exploratory prioritisation and transparency. Variables directly used to define the screening-derived abnormality-burden outcome were excluded from the predictor set. The analysis used a held-out testing strategy, with imputation parameters estimated within the training data and then applied to the test data to reduce information leakage. Logistic regression, ridge logistic regression, random forest, and XGBoost models were examined, and model performance was evaluated using discrimination, calibration, and threshold-based metrics.

Because these models were exploratory, internally assessed, not externally validated, and showed only modest discrimination, they were not used to support the primary inference and were not interpreted as diagnostic, prognostic, or deployment-ready screening tools. Predictor transparency, internal prediction performance, calibration, threshold-based metrics, and ROC results are reported in the Supplementary Material.

All analyses were performed using R version 4.5.0. Modified Poisson models were fitted using Poisson regression with a log link, and robust HC3 standard errors were estimated using sandwich-based variance estimators. Multiple imputation was performed using chained equations with 20 imputed datasets and 10 iterations per imputation; estimates were pooled using Rubin’s rules. The main analytical workflow used the following R packages: data.table, dplyr, tidyr, stringr, purrr, readr, openxlsx, broom, sandwich, lmtest, tibble, mice, MASS, pROC, PRROC, glmnet, randomForest, and xgboost.

The manuscript was prepared with reference to STROBE reporting principles for cross-sectional observational studies (29). A two-sided *p* value <0.05 was considered statistically significant for the primary analyses. Supplementary analyses were interpreted in conjunction with effect estimates, confidence intervals, direction, magnitude, and consistency across model specifications rather than isolated *p* values.

## 3 Results

### 3.1 Sample characteristics, missingness, and exposure distribution

After data harmonisation, quality control, and restriction to women, 1,163 older women were included in the final analytic sample. High screening-derived abnormality burden was present in 320 participants, corresponding to a prevalence of 27.52%. Diabetes-related exposure was present in 262 participants, corresponding to a prevalence of 22.53%.

Based on the cohort-specific labour-type physical-activity index, 1,131 participants had complete three-category physical-activity classification. Among them, 432 women were classified as Inactive, 143 as LowActive, and 556 as HighActive. The median physical-activity index among active participants was 180. Missingness was low across variables used in the primary models, with the highest missing rate observed for three-category labour-type physical-activity classification (32/1,163, 2.75%). The screening-derived abnormality-burden outcome and diabetes-related exposure had no missing values.

The difference between the final analytic sample size and model-specific sample sizes was attributable to exposure or covariate missingness rather than outcome missingness. Primary regression models using the three-category labour-type physical-activity variable included 1,131 women, whereas the BMI-adjusted sensitivity model included 1,129 women. Diabetes-related exposure was similarly distributed across the three labour-type physical-activity categories: 21.99% in the Inactive group, 22.38% in the LowActive group, and 22.48% in the HighActive group. Diabetes-related exposure was therefore similarly distributed across labour-type physical-activity strata (Supplementary Tables S2, S11).

### 3.2 Baseline characteristics by high screening-derived abnormality-burden status

Compared with women without high abnormality burden, women with high screening-derived abnormality burden showed a less favourable metabolic and renal–urinary screening profile. The high-abnormality-burden group had higher BMI, fasting plasma glucose, triglycerides, creatinine, and urea, and lower HDL-C. Diabetes-related exposure was also more common in the high-abnormality-burden group than in the non-high-abnormality-burden group (33.12% vs. 18.51%). Hypertension, polypharmacy, and urinalysis occult blood positivity were also more frequent among women with high abnormality burden.

In contrast, the binary inactivity indicator did not differ significantly between the high-abnormality-burden and non-high-abnormality-burden groups. These descriptive findings indicate that high screening-derived abnormality burden was more clearly characterised by metabolic, renal–urinary, and medication-related screening abnormalities than by a simple active–inactive contrast. Because several variables in Table 1 were components or component-related measures of the abnormality-burden outcome, between-group comparisons should be interpreted descriptively rather than as independent explanatory or etiological factors for high abnormality burden.

**Table 1:** Baseline characteristics of older women by high screening-derived abnormality-burden status.

Variable	Overall	Non-high abnormality burden	High abnormality burden	*p*-value
Age, years	72.55 ± 5.88	72.69 ± 6.00	72.16 ± 5.52	0.148
BMI, kg/m^2^	24.50 ± 3.38	24.09 ± 3.11	25.58 ± 3.80	<0.001
SBP, mmHg	145.47 ± 20.40	145.54 ± 20.06	145.28 ± 21.32	0.852
DBP, mmHg	85.37 ± 10.70	85.06 ± 10.63	86.20 ± 10.86	0.109
FPG, mmol/L	6.11 ± 1.60	5.96 ± 1.44	6.53 ± 1.88	<0.001
TG, mmol/L	2.04 ± 1.17	1.80 ± 0.99	2.66 ± 1.37	<0.001
HDL-C, mmol/L	1.41 ± 0.36	1.50 ± 0.36	1.17 ± 0.23	<0.001
Creatinine, μmol/L	71.04 ± 19.31	69.38 ± 16.07	75.40 ± 25.52	<0.001
Urea, mmol/L	6.33 ± 1.78	6.11 ± 1.62	6.92 ± 2.04	<0.001
Diabetes-related exposure, *n* (%)	262 (22.53)	156 (18.51)	106 (33.12)	<0.001
Hypertension, *n* (%)	585 (50.30)	405 (48.04)	180 (56.25)	0.012
Inactive under binary labour-type PA robustness definition, *n* (%)	432 (38.20)	320 (39.02)	112 (36.01)	0.352
Polypharmacy, *n* (%)	45 (3.87)	18 (2.14)	27 (8.44)	<0.001
Urinalysis occult blood positivity, *n* (%)	421 (36.20)	221 (26.22)	200 (62.50)	<0.001

Values are presented as mean ± standard deviation or n (%). *p*-values were derived from Student’s *t*-test for continuous variables and chi-square tests for categorical variables and were used for descriptive comparison only. High screening-derived abnormality burden was defined as the presence of three or more abnormal components among routinely collected non-diagnostic screening indicators. This internally derived outcome was not intended to diagnose constipation, estimate constipation prevalence, replace symptom-based bowel assessment, function as a validated frailty index, or represent a validated multidomain geriatric phenotype. Percentages were calculated using available non-missing data for each variable. The binary labour-type PA variable shown in this table was used for descriptive and robustness purposes only; the primary PA exposure in regression models was the three-category labour-type PA variable. Between-group comparisons are descriptive; variables that were components or component-related measures of the abnormality-burden outcome should not be interpreted as independent explanatory or etiological factors for high abnormality burden.

### 3.3 Composition of the screening-derived abnormality-burden outcome

The screening-derived abnormality-burden outcome captured clustering of routine screening abnormalities rather than symptom-defined constipation or a validated multidomain geriatric phenotype. Among the seven component indicators, elevated triglycerides, low HDL-C, urinalysis occult blood positivity, and renal abnormality were relatively common, with positive proportions of 51.47, 43.13, 36.20, and 31.35%, respectively. Abnormal BMI was present in 15.76% of participants, whereas polypharmacy and functional impairment were less frequent, with positive proportions of 3.87 and 3.44%, respectively.

These distributions indicate that the abnormality-burden outcome was shaped mainly by metabolic and renal–urinary components, with smaller contributions from medication-related and functional components. This uneven component distribution was an important feature of the available routine screening data and was considered in the component-influence and sensitivity analyses described below (Supplementary Table S3).

### 3.4 Primary diabetes-related association and secondary contextual labour-type physical-activity estimates

In modified Poisson regression models with robust HC3 standard errors, diabetes-related exposure was consistently associated with high screening-derived abnormality burden. In the crude model, diabetes-related exposure was associated with a higher prevalence of high abnormality burden (PR 1.751, 95% CI 1.448–2.119). The association remained stable after adjustment for age (PR 1.739, 95% CI 1.436–2.108).

In the primary adjusted model without BMI, diabetes-related exposure remained associated with high abnormality burden (PR 1.668, 95% CI 1.367–2.035). Because abnormal BMI was one component of the screening-derived abnormality-burden outcome, continuous BMI was not included in the primary adjusted model. In the BMI-adjusted sensitivity model, the association remained similar (PR 1.614, 95% CI 1.329–1.961).

**Figure 2:**
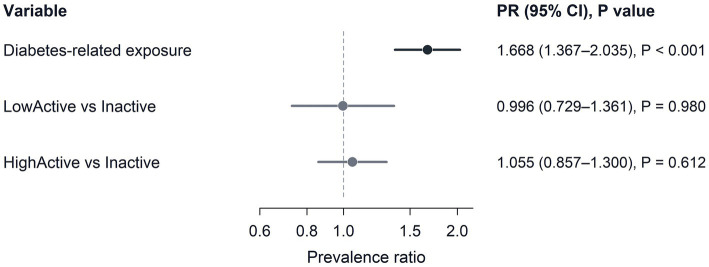
Primary adjusted diabetes-related association and secondary contextual labour-type physical-activity estimates for high screening-derived abnormality burden. Forest plot of prevalence ratios and 95% confidence intervals for diabetes-related exposure, LowActive, and HighActive from the primary adjusted modified Poisson regression model with robust HC3 standard errors. Inactive was the reference category for labour-type physical activity. The model adjusted for age, hypertension, and current smoking. Continuous BMI was not included because abnormal BMI was one component of the screening-derived abnormality-burden outcome. The vertical dashed line indicates PR = 1.

In contrast, neither LowActive nor HighActive was independently associated with high abnormality burden in any model. In the primary adjusted model without BMI, the PR was 0.996 for LowActive versus Inactive (95% CI 0.729–1.361) and 1.055 for HighActive versus Inactive (95% CI 0.857–1.300). These findings indicate that diabetes-related exposure was associated with high screening-derived abnormality burden. The available cohort-specific labour-type physical-activity measure did not independently distinguish abnormality-burden status and was interpreted as a secondary contextual finding rather than as the primary conceptual contribution of the manuscript (Table 2; Figure 2).

**Table 2:** Primary diabetes-related association and secondary contextual labour-type physical-activity estimates for high screening-derived abnormality burden.

Model	Term	PR	95% CI	*p*-value	*N*
Model 1: crude	Diabetes-related exposure	1.751	1.448–2.119	<0.001	1,131
Model 1: crude	LowActive vs. inactive	1.049	0.771–1.428	0.759	1,131
Model 1: crude	HighActive vs. inactive	1.106	0.902–1.357	0.331	1,131
Model 2: age-adjusted	Diabetes-related exposure	1.739	1.436–2.108	<0.001	1,131
Model 2: Age-adjusted	LowActive vs. inactive	1.024	0.749–1.399	0.883	1,131
Model 2: age-adjusted	HighActive vs. Inactive	1.074	0.873–1.321	0.502	1,131
Model 3: primary adjusted without BMI	Diabetes-related exposure	1.668	1.367–2.035	<0.001	1,131
Model 3: primary adjusted without BMI	LowActive vs. inactive	0.996	0.729–1.361	0.980	1,131
Model 3: primary adjusted without BMI	HighActive vs. inactive	1.055	0.857–1.300	0.612	1,131
Model 4: BMI-adjusted sensitivity	Diabetes-related exposure	1.614	1.329–1.961	<0.001	1,129
Model 4: BMI-adjusted sensitivity	LowActive vs. inactive	0.966	0.716–1.302	0.819	1,129
Model 4: BMI-adjusted sensitivity	HighActive vs. inactive	1.037	0.846–1.271	0.729	1,129

PR, prevalence ratio; CI, confidence interval; BMI, body mass index. Modified Poisson regression with robust HC3 standard errors was used. Inactive was the reference category for the three-category labour-type PA variable. Model 1 was unadjusted. Model 2 adjusted for age. Model 3 was the primary adjusted model and included age, hypertension, and current smoking. Continuous BMI was not included in Model 3 because abnormal BMI was one component of the screening-derived abnormality-burden outcome. Model 4 additionally adjusted for continuous BMI as a sensitivity analysis. Effective sample size varied slightly across models because complete-case modelling was used. Only the primary exposure terms are shown; covariates included in each model are specified in the Note.

### 3.5 Component-influence, metabolic-component sensitivity, robustness, and exploratory prediction analyses

Several sensitivity analyses were conducted to examine whether the main diabetes-related association was robust to alternative outcome definitions, component composition, metabolic-component influence, exposure definitions, missing-data handling, and additional covariate adjustment. These analyses were interpreted as component-influence, metabolic-component sensitivity, and robustness analyses rather than as validation of the internally derived abnormality-burden outcome.

First, the binary labour-type physical-activity robustness analysis yielded a pattern consistent with the primary three-category analysis. Diabetes-related exposure remained associated with high abnormality burden, whereas Active versus Inactive status was not independently associated with high abnormality burden (Supplementary Table S4).

Second, alternative-threshold analyses showed that the diabetes-related association remained positive when high abnormality burden was defined using either a broader threshold of ≥2 abnormal components or a stricter threshold of ≥4 abnormal components. In the BMI-adjusted sensitivity models, the PR was 1.147 (95% CI 1.038–1.269) for the ≥2 threshold and 2.392 (95% CI 1.720–3.328) for the ≥4 threshold. Labour-type physical-activity categories remained non-significant across threshold definitions (Supplementary Table S5).

Third, analyses addressing BMI-component overlap showed similar diabetes-related exposure estimates across the primary adjusted model without BMI, the BMI-adjusted sensitivity model, and the model recalculating the abnormality-burden score after excluding the BMI-abnormality component. After excluding the BMI-abnormality component, diabetes-related exposure remained associated with high abnormality burden (PR 1.696, 95% CI 1.353–2.127) (Table 4; Supplementary Table S6).

**Table 4:** Summary of key component-influence, metabolic-component sensitivity, and robustness analyses.

Sensitivity analysis	Outcome/definition	Adjustment strategy	Diabetes-related exposure PR	95% CI	*p*-value	Result summary
BMI-adjusted sensitivity	Original high abnormality burden, ≥3 components	Age, hypertension, BMI, current smoking	1.614	1.329–1.961	<0.001	Association remained after BMI adjustment
Excluding BMI-abnormality component	Abnormality-burden score recalculated after removing abnormal BMI	Age, hypertension, BMI, current smoking	1.696	1.353–2.127	<0.001	Association remained after excluding the BMI component
Non-metabolic abnormality burden	Excluding elevated triglycerides, low HDL-C, and abnormal BMI	Age, hypertension, current smoking	1.463	1.077–1.988	0.015	Association persisted in the non-metabolic abnormality-burden analysis
Leave-one-component-out analyses	Each of the seven components removed one at a time	Age, hypertension, current smoking	1.497–2.258	All 95% CIs excluded 1.000	All *p* < 0.001	Association remained across component-exclusion analyses
Alternative diabetes definition without medication use	Self-reported diabetes or FPG ≥ 7.0 mmol/L	Age, hypertension, current smoking	1.686	1.382–2.058	<0.001	Association remained after excluding medication-based exposure definition
MICE pooled BMI-adjusted analysis	Original high abnormality burden, imputed full sample	Age, hypertension, BMI, current smoking	1.577	1.301–1.912	<0.001	Directionally consistent after multiple imputation
Additional covariate-adjusted model	Original high abnormality burden with additional routinely available screening covariates	Age, hypertension, BMI, current smoking, SBP, DBP, ultrasound abnormality, cognitive impairment	1.639	1.349–1.992	<0.001	Association remained after additional covariate adjustment

PR, prevalence ratio; CI, confidence interval; BMI, body mass index; HDL-C, high-density lipoprotein cholesterol; FPG, fasting plasma glucose; MICE, multiple imputation by chained equations; SBP, systolic blood pressure; DBP, diastolic blood pressure. Modified Poisson regression with robust HC3 standard errors was used. This table summarises selected sensitivity analyses addressing component influence, metabolic-component dominance, BMI-related sensitivity, non-metabolic abnormality burden, diabetes-exposure definition, missing-data handling, and additional covariate adjustment. The displayed estimates refer to diabetes-related exposure. Detailed results are provided in Supplementary Tables S6–S11, S13. For the leave-one-component-out analyses, the displayed PR range represents diabetes-related exposure estimates across the seven component-exclusion models; full component-specific results are provided in Supplementary Table S9.

Fourth, as a key interpretive sensitivity analysis addressing potential overlap between diabetes-related exposure and metabolic outcome components, a non-metabolic abnormality-burden analysis was conducted after excluding elevated triglycerides, low HDL-C, and abnormal BMI. Diabetes-related exposure remained associated with high non-metabolic abnormality burden in the primary adjusted model without BMI (PR 1.463, 95% CI 1.077–1.988). Labour-type physical-activity categories remained non-significant (Table 4; Supplementary Table S7).

Fifth, domain-specific analyses showed heterogeneous patterns across domains. Diabetes-related exposure was associated with metabolic burden and medication–functional burden, but not with renal–urinary burden. Because medication–functional burden was relatively infrequent and the number and prevalence of components differed across domains, these domain-specific findings were interpreted as exploratory analyses of component contribution and outcome consistency rather than as evidence of domain-specific mechanisms (Supplementary Table S8).

Sixth, leave-one-component-out analyses showed that the association between diabetes-related exposure and high abnormality burden remained across models that removed each component from the abnormality-burden outcome one at a time. Across the seven component-exclusion models, diabetes-related exposure estimates remained statistically significant, with PRs ranging from 1.497 to 2.258 (Table 4; Supplementary Table S9).

Seventh, sensitivity analyses using alternative diabetes-related exposure definitions yielded consistent findings. The association remained significant when glucose-lowering medication use was excluded from the exposure definition (PR 1.686, 95% CI 1.382–2.058), when fasting plasma glucose ≥7.0 mmol/L alone was used, and when self-reported diabetes alone was used (Table 4; Supplementary Table S10).

Eighth, missing-data analyses yielded results directionally consistent with the complete-case models. In the MICE pooled BMI-adjusted model, diabetes-related exposure remained associated with high abnormality burden (PR 1.577, 95% CI 1.301–1.912), whereas LowActive and HighActive remained non-significant (Table 4; Supplementary Table S11).

Ninth, analyses treating the abnormality-burden outcome as an ordered categorical outcome or as a count-based score also produced consistent patterns. Diabetes-related exposure was associated with higher abnormality burden in both models, whereas labour-type physical-activity categories remained non-significant (Supplementary Table S12).

Finally, additional adjustment for routinely available screening variables, including systolic blood pressure, diastolic blood pressure, ultrasound abnormality, and cognitive impairment, did not materially alter the diabetes-related association (PR 1.639, 95% CI 1.349–1.992). Labour-type physical-activity categories remained non-significant in this additional covariate-adjusted model (Table 4; Supplementary Table S13).

Supplementary leakage-free internal prediction analyses showed only modest discrimination across models, with AUC values ranging from 0.527 to 0.595 and an optimism-corrected AUC of 0.587 for the logistic model. These analyses were retained only for transparency and exploratory internal prioritisation. They were not used to support diagnostic, prognostic, or deployment-ready claims. Predictor transparency, model performance, threshold-based metrics, and calibration information are provided in Supplementary Table S14 and Supplementary Figures S1–S3.

### 3.6 Supplementary descriptive stratified pattern according to diabetes-related exposure and binary labour-type physical activity

A descriptive stratified analysis was conducted according to diabetes-related exposure and the binary labour-type physical-activity classification. This analysis was retained for transparency because labour-type physical activity was available in the routine examination platform and had been used in previous analyses from the same parent database. The pattern showed a higher prevalence of high screening-derived abnormality burden among women with diabetes-related exposure than among those without diabetes-related exposure, whereas differences by binary labour-type physical-activity status were smaller. This analysis was descriptive only and was not interpreted as evidence of causal interaction, a primary physical-activity effect, or a repetition of previous labour-focused analyses. Detailed stratum-specific prevalence estimates are provided in Supplementary Table S15 and Supplementary Figure S4.

## 4 Discussion

### 4.1 Principal findings

In this community-based cross-sectional study of older women in rural Northeast China, high screening-derived abnormality burden was common in a symptom-unavailable primary-care screening setting. Diabetes-related exposure was associated with a higher prevalence of high abnormality burden across crude, age-adjusted, primary adjusted, and multiple sensitivity analyses. Importantly, the association also remained in the non-metabolic abnormality-burden analysis, suggesting that the observed diabetes-related association was not fully explained by shared metabolic components alone. Labour-type physical activity did not independently distinguish abnormality-burden status, but this finding was interpreted as secondary and contextual because the available activity measure was crude, cohort-specific, and routine-record based.

The main contribution of this study is not the identification of constipation, bowel disease, or a validated geriatric phenotype, but the evaluation of a pragmatic screening framework based on routinely available primary-care examination indicators. The outcome should therefore be interpreted as an internally derived screening-derived abnormality-burden outcome rather than as symptom-defined constipation, bowel dysfunction, a validated multidomain geriatric vulnerability phenotype, a validated frailty index, a comprehensive geriatric assessment score, or a clinical gastrointestinal diagnosis. Because the routine examination platform did not include Rome criteria, stool-frequency records, stool-form assessment, stool diaries, or clinician-adjudicated gastrointestinal evaluation, the findings should be understood as evidence of clustered routine screening abnormalities rather than evidence of a formal bowel-disease endpoint (8, 9).

### 4.2 Diabetes-related exposure and screening-derived abnormality burden

The association between diabetes-related exposure and high abnormality burden is consistent with a systemic primary-care screening framework. In older adults, glycaemic burden often coexists with adverse metabolic profiles, renal–urinary abnormalities, treatment complexity, and functional limitations, all of which may be reflected in clustered routine screening abnormalities (13, 30). In the present study, diabetes-related exposure remained associated with high abnormality burden after BMI adjustment, after excluding the BMI-abnormality component, after using a non-metabolic abnormality-burden outcome, after applying alternative diabetes definitions, after multiple imputation, and after additional covariate adjustment. This pattern suggests that the diabetes-related association was not explained solely by BMI overlap, medication-based exposure classification, missing-data handling, or shared metabolic components alone.

These findings support the interpretation of diabetes-related exposure as a practical marker of screening-derived abnormality burden in older women. However, they should not be interpreted as evidence that diabetes-related exposure causes constipation, bowel dysfunction, or any specific gastrointestinal disease process. Although diabetic autonomic neuropathy and gastrointestinal dysmotility are biologically relevant in bowel-specific research, these pathways were not measured in the present dataset and should therefore be treated only as background biological plausibility rather than demonstrated mechanisms (15, 31, 32). Given the cross-sectional design and the absence of symptom-based bowel measures, the observed association is best interpreted as co-occurrence between diabetes-related glycaemic burden and clustered routinely observable metabolic, renal–urinary, medication-related, and functional screening abnormalities, rather than as evidence of a causal or disease-specific pathway.

### 4.3 Secondary contextual interpretation of labour-type physical activity

The null association observed for labour-type physical activity should be interpreted as a secondary contextual finding rather than as the primary contribution of the present study. The available activity variable was derived from routine records of weekly frequency and session duration and did not capture activity intensity, leisure-time exercise dose, occupational workload, activity domain, or total energy expenditure (7, 22). Therefore, it should be interpreted as a crude, cohort-specific labour-type activity indicator rather than as a validated exercise-dose measure, standardised physical-activity assessment, or standard health-promoting physical-activity indicator.

In rural older women, recorded activity may represent agricultural labour, domestic responsibility, seasonal work, and continued work obligation rather than structured or discretionary exercise (20, 21). Health-related selection is also possible in a cross-sectional design: women with greater health burden may reduce activity, whereas others may continue labour-related activity because of household or agricultural necessity. Substantial measurement imprecision and exposure misclassification may therefore have attenuated potential associations between physical activity and abnormality-burden status. Thus, the null finding applies only to this cohort-specific labour-type activity measure and should not be generalised to objectively measured physical activity, leisure-time exercise, or structured exercise interventions. This interpretation preserves the rural screening context while distinguishing the present study from previous labour-focused analyses.

### 4.4 Interpretation of the screening-derived abnormality-burden outcome

The central interpretive issue in this study is the appropriate boundary of the screening-derived abnormality-burden outcome. This outcome was not designed to diagnose constipation, estimate constipation prevalence, replace symptom-based bowel assessment, function as a validated frailty index, or serve as a comprehensive geriatric assessment score. Symptom-defined bowel outcomes require bowel-specific information such as Rome criteria, stool frequency, stool form, stool diaries, or clinician-adjudicated gastrointestinal assessment, which were not available in the present routine health-examination platform (8, 9). Similarly, validated frailty indices and comprehensive geriatric assessment frameworks require broader and more systematically measured multidimensional health information than was available in this screening dataset (10, 33). Therefore, the present outcome should be interpreted as an internally derived, unweighted, exploratory summary of routinely observable screening abnormalities, not as a clinical diagnosis, a validated geriatric syndrome, or a bowel-specific endpoint.

The seven components were selected to represent routinely recorded abnormalities across metabolic, renal–urinary, medication-related, and functional domains. The metabolic components, including triglycerides, HDL-C, and BMI abnormality, reflected cardiometabolic screening burden that may cluster with diabetes-related exposure and broader ageing-related vulnerability (34). The renal–urinary components reflected renal or urinary screening abnormalities that may indicate systemic burden and the need for further clinical review in older adults, although they were not bowel-specific markers (35). Polypharmacy was included as a marker of treatment complexity and medication-review need in older adults; the use of five or more medications is also a commonly used operational definition of polypharmacy in geriatric research (25). Functional impairment was included because ADL limitation and disability are core indicators of reduced functional reserve and self-management capacity in older adults (36). These components support the use of the outcome as an exploratory primary-care abnormality-burden summary, while reinforcing that it should not be interpreted as a bowel-health endpoint or validated geriatric phenotype.

The component distribution further supports cautious interpretation. The abnormality-burden outcome was shaped mainly by metabolic and renal–urinary abnormalities, whereas medication-related and functional components were infrequent. This uneven distribution limits any claim that the score represents a balanced multidomain geriatric syndrome or externally validated clinical phenotype. Rather than assuming that all domains contributed equally, we treated this imbalance as an empirical feature of the available primary-care screening data and examined it through component-influence, metabolic-component sensitivity, and robustness analyses.

Several analyses were used to examine robustness and component-related interpretation. The diabetes-related association remained after BMI adjustment, after excluding the BMI-abnormality component, and after using a non-metabolic abnormality-burden outcome that excluded triglycerides, HDL-C, and BMI abnormality. Leave-one-component-out analyses also showed that the association remained across models removing each component one at a time. Domain-specific analyses showed heterogeneous patterns across domains; because the number and prevalence of components differed substantially across domains, these findings were interpreted only as exploratory evidence of component contribution and outcome consistency, rather than as evidence of domain-specific mechanisms.

Taken together, these analyses support a cautious screening-oriented interpretation. The outcome captures clustered routine screening abnormalities observable in primary-care data, but it does not establish external validity against symptom-based bowel outcomes, validated frailty measures, comprehensive geriatric assessment, healthcare utilisation, or prospective clinical events. This distinction is important: the outcome may help identify individuals who warrant further assessment, but it should not be treated as a substitute for clinical diagnosis, symptom-based bowel assessment, validated frailty assessment, or comprehensive geriatric evaluation.

### 4.5 Rural Northeast China context and relation to previous literature

The rural Northeast China setting is important for interpreting these findings. In cold-climate agricultural communities, later-life activity may reflect seasonal labour, household responsibilities, environmental constraints, and continued work obligation rather than discretionary leisure-time exercise (7). At the same time, routine primary-care examination systems more readily capture metabolic, renal–urinary, medication-related, and functional screening abnormalities than detailed bowel symptoms (4). These contextual features may partly explain why the clearest association in the present study was related to diabetes-related exposure and screening-derived abnormality burden rather than to the labour-type activity indicator.

The present study should also be distinguished from two previous analyses using the same parent health-examination database (7, 22). The previous hypertension analysis examined labour-type physical activity and alcohol use in relation to measured hypertension in the overall older-adult population. The previous labour–diabetes analysis examined occupational/labour-type physical activity in relation to total diabetes burden, the triglyceride–glucose index, and urine occult blood positivity in the overall older-adult population. In contrast, the present study restricted the analytic population to older rural women and used cumulative screening-derived abnormality burden as the primary outcome. It did not use hypertension, total diabetes burden, the triglyceride–glucose index, or urine occult blood positivity as standalone primary endpoints. Accordingly, the current manuscript should be interpreted as a female-specific primary-care screening analysis rather than as a repetition of the previous labour, hypertension, diabetes, TyG, or UOB-focused analyses.

Although diet and caregiving information were not directly measured, the regional context of rural Northeast China—including cold winters, seasonal reductions in outdoor activity, possible regional dietary patterns, and household or caregiving responsibilities among older women—may shape both metabolic screening abnormalities and the meaning of recorded labour-type activity. Because these contextual factors were not directly measured in the present dataset, they should be interpreted as background considerations and should be assessed directly in future studies.

### 4.6 Aging and public-health implications for research, practice, education, and policy

The implications of this study extend beyond clinical follow-up and are directly relevant to aging and public health. At the research level, the study demonstrates how routinely collected older-adult health-examination data can be translated into a transparent, bounded screening-burden framework for an underserved older female population, while also identifying the validation needed before wider use. At the practice level, diabetes-related exposure may serve as a readily available flag during annual older-adult health examinations to prompt structured review of clustered abnormalities, medication burden, functional status, health-guidance needs, and referral priorities. At the education level, community physicians, nurses, village doctors, and public-health workers could be trained to interpret clustered routine abnormalities as whole-person older-adult risk signals rather than as isolated laboratory deviations or disease-specific diagnoses. At the policy and programme-planning level, the findings support pilot testing of stepped follow-up pathways within rural older-adult health-management systems, especially in cold-climate agricultural communities where seasonality, continued labour demands, and access constraints may shape healthy aging.

Within this public-health pathway, older rural women with diabetes-related exposure and clustered routine screening abnormalities may warrant additional attention during routine older-adult health examinations. Such attention could include brief bowel-symptom inquiry, medication review, hydration and dietary counselling, functional assessment, and referral when clinically indicated (8, 9, 25, 33). These steps should be understood as screening, triage, and follow-up assessment strategies rather than diagnosis of constipation, evidence of bowel disease, or proof of intervention efficacy.

The study also illustrates how routine primary-care data can be used cautiously and transparently for research translation. In settings where symptom-rich assessment is unavailable, screening-derived abnormality-burden measures may support prioritisation for further review. Their role, however, should remain clearly separated from clinical diagnosis. They may help identify individuals who need additional assessment, but they cannot replace symptom-based bowel evaluation, comprehensive geriatric assessment, or clinician judgement (8–10, 33).

Future research should proceed in three directions. First, symptom-based bowel-health measures, including stool frequency, stool form, Rome criteria, and clinician-adjudicated bowel outcomes, should be incorporated to examine whether the screening-derived abnormality-burden outcome is associated with formal bowel outcomes. Second, longitudinal studies are needed to clarify temporality and evaluate whether diabetes-related exposure predicts persistence, progression, healthcare use, or clinical consequences of screening-derived abnormality burden. Third, more refined physical-activity assessment should distinguish labour-type activity from structured health-promoting exercise by incorporating intensity, duration, domain, and energy expenditure (16, 18, 20, 21). Before wider public-health implementation, this screening framework should be externally validated and evaluated within feasible, equitable, and workforce-supported older-adult health-management pathways.

### 4.7 Strengths and limitations

This study has several strengths. It used real-world annual health-examination data from an underrepresented cold-climate rural setting and focused on older women, a population in whom metabolic burden, treatment complexity, and functional limitation may cluster. The study used an interpretable prevalence-ratio framework and provided transparent operational definitions and interpretive boundaries for the screening-derived abnormality-burden outcome. Several sensitivity analyses addressed key sources of interpretive concern, including BMI overlap, metabolic-component dominance, single-component dependence, non-metabolic abnormality burden, alternative diabetes definitions, additional covariate adjustment, and missing-data handling. The supplementary internal prediction analysis was deliberately separated from the primary inference and interpreted only as exploratory transparency evidence.

Several limitations should also be acknowledged. First, the cross-sectional design precludes causal inference, and the observed associations may reflect bidirectional relationships, common-cause pathways, or residual confounding. Second, the outcome was an internally derived screening-derived abnormality-burden outcome rather than a symptom-based constipation diagnosis, validated frailty index, comprehensive geriatric assessment score, or externally validated multidomain geriatric phenotype. The data did not include Rome IV criteria, stool frequency, stool form, stool diaries, or clinician-adjudicated bowel outcomes (8, 9). Third, the labour-type physical-activity variable was derived from routine records and did not capture intensity, domain-specific workload, leisure-time exercise, occupational load, or energy expenditure (16, 18, 20, 21). It was therefore interpreted as a secondary contextual variable rather than as the primary conceptual exposure of the present study. Fourth, residual confounding remains possible because diet quality, hydration, depressive symptoms, detailed medication classes, bowel symptoms, and objectively measured physical activity were not available. Fifth, diabetes-related exposure was based on routine screening information and did not include repeated confirmatory testing, diabetes duration, glycaemic history, or adjudicated complication status (12). Sixth, the study was conducted within a single regional primary-care screening platform, and external transportability should be assessed cautiously. Finally, the internal prediction analyses showed only modest discrimination and were not externally validated; they should not be interpreted as diagnostic, prognostic, or deployment-ready prediction models.

Overall, the findings support a cautious, screening-oriented interpretation. In older rural women, diabetes-related exposure was associated with screening-derived abnormality burden. Labour-type physical activity did not show an independent association under the available crude, cohort-specific measurement framework, but this result was interpreted only as a secondary contextual finding. The outcome should not be interpreted as constipation, bowel dysfunction, a validated frailty index, a comprehensive geriatric assessment score, or a validated multidomain geriatric phenotype (10, 33). Instead, it may help identify older women who warrant more detailed symptom inquiry, medication review, functional assessment, and broader clinical evaluation in routine primary-care settings.

## 5 Conclusion

In older rural women from Northeast China, diabetes-related exposure was associated with a higher prevalence of screening-derived abnormality burden. This internally derived outcome should be interpreted as a pragmatic primary-care screening indicator of clustered routine screening abnormalities, not as symptom-defined constipation, bowel dysfunction, a validated frailty index, a comprehensive geriatric assessment score, or a validated multidomain geriatric phenotype. Labour-type physical activity did not independently distinguish abnormality-burden status under the available crude routine-record measurement framework, but this result should be interpreted as a secondary contextual finding rather than as the primary contribution of the study. In routine older-adult health examinations, this screening-oriented framework may help identify women who warrant more detailed symptom inquiry, medication review, functional assessment, and individualised health guidance. Longitudinal studies incorporating symptom-based bowel measures, external validation against clinical or geriatric outcomes, and more refined contextual activity assessment are needed before broader clinical or public-health implementation. At the aging-and-public-health level, this framework may help existing older-adult health-examination systems prioritise community follow-up for underserved rural women with clustered screening abnormalities, thereby linking diabetes-related chronic-disease management with healthy-aging support and integrated care. Such use should be framed as population-level risk recognition, workforce-supported health guidance, and referral prioritisation rather than as clinical diagnosis, validated prognostic scoring, or evidence of intervention effectiveness.

## Data Availability

The raw data supporting the conclusions of this article will be made available by the authors, without undue reservation.
