# Supplementary material for: Diabetes-related exposure and screening-derived abnormality burden among older rural women in Northeast China: a secondary analysis with contextual labour-type physical activity assessment
Source: Front Public Health. 2026 Jul 1;14:1841622. doi: 10.3389/fpubh.2026.1841622 (PMC13371314; doi:10.3389/fpubh.2026.1841622)
Supplement: Supplementary file 1 [file Table_1.DOCX]

**Supplementary Table S1. Conceptual rationale and interpretive boundary for components of the screening-derived abnormality-burden outcome**

| **Domain** | **Component** | **Operational definition / meaning in routine screening** | **Conceptual rationale for inclusion** | **Interpretive boundary** |
| --- | --- | --- | --- | --- |
| Metabolic burden | Elevated triglycerides | Elevated triglycerides, operationalised as triglycerides ≥1.7 mmol/L in the present analysis. | Elevated triglycerides were included as a routinely available marker of metabolic dysregulation and cardiometabolic screening burden. In older adults, lipid abnormalities may cluster with diabetes-related exposure, multimorbidity, renal risk, and broader physiological burden. Within an accumulation-of-deficits and abnormality-burden screening framework, this component was used to represent metabolic burden rather than bowel-specific pathology [10–13,30,34]. | This component is not bowel-specific and should not be interpreted as evidence of constipation, bowel dysfunction, or gastrointestinal disease. Its inclusion reflects metabolic screening burden only. |
| Metabolic burden | Low HDL-C | Low HDL-C, operationalised as HDL-C <1.3 mmol/L in the present analysis. | Low HDL-C was included as a routinely measured indicator of adverse cardiometabolic status. Together with triglyceride abnormality and BMI abnormality, it helps characterise the metabolic domain of the abnormality-burden outcome. Its purpose was to capture clustered metabolic screening burden observable in primary-care examinations, not to identify bowel symptoms or bowel disease [10–13,30,34]. | This component is not bowel-specific and does not diagnose constipation, bowel dysfunction, or bowel disease. It should be interpreted only as part of the metabolic screening domain. |
| Metabolic burden | Abnormal BMI | Abnormal BMI, operationalised as BMI <18.5 kg/m² or BMI ≥28 kg/m², with the obesity threshold aligned with Chinese adult BMI classification. | Abnormal BMI was included to capture body-composition and nutritional/metabolic screening burden. Both underweight and obesity may reflect altered physiological reserve, metabolic dysregulation, or broader health risk in older adults. In this outcome, BMI abnormality was treated as a routinely available screening marker within an abnormality-burden framework, and potential overlap with model adjustment was examined in sensitivity analyses [1,6,10,11,24,33]. | This component is not a bowel-symptom measure and should not be interpreted as a constipation indicator, frailty diagnosis, or comprehensive geriatric assessment measure. |
| Renal–urinary burden | Renal abnormality | Renal abnormality, operationalised as creatinine >84 μmol/L and/or urea >7.1 mmol/L according to routine laboratory coding in the examination dataset. | Renal abnormality was included as a routinely available marker of renal–systemic screening burden. Renal impairment may coexist with diabetes-related exposure, multimorbidity, medication complexity, and reduced physiological reserve in older adults. In the present outcome, this component was used to identify broader need for clinical review rather than to infer a bowel-specific mechanism [10,11,13,30,35]. | This component is not bowel-specific and does not indicate constipation, bowel dysfunction, or gastrointestinal disease. It represents renal–systemic screening burden only. |
| Renal–urinary burden | Urinalysis occult blood positivity | Urinalysis occult blood positivity, operationalised as trace or greater occult-blood positivity, positive coding, or equivalent positive coding on routine urinalysis. | Urinalysis occult blood positivity was included as a routine renal–urinary screening abnormality that may indicate the need for further medical assessment in older adults. Although non-specific, it contributes to the renal–urinary domain of the abnormality-burden outcome and reflects abnormalities detectable during primary-care health examinations [10,11,30,35]. | This component is not bowel-specific and should not be used as a bowel-health, constipation, gastrointestinal, or disease-specific diagnostic marker. |
| Medication-related burden | Polypharmacy | Polypharmacy, operationalised as an existing polypharmacy flag in the routine record or concurrent use of ≥5 medications when medication-count information was available. | Polypharmacy was included as a marker of treatment complexity and possible multimorbidity burden. In older adults, medication burden may reflect chronic disease accumulation, adverse-event risk, and need for medication review. Some medications may affect bowel function, but the present dataset did not capture medication classes in sufficient detail; therefore, polypharmacy was used as a general treatment-complexity marker within the abnormality-burden outcome [2,10,11,25,30]. | This component does not identify specific constipation-inducing medication classes and is not a direct measure of bowel symptoms, constipation, or bowel dysfunction. It should be interpreted as medication-related screening burden only. |
| Functional limitation | Functional impairment | Functional impairment, operationalised as ADL impairment and/or recorded disability in the annual health-examination platform. | Functional impairment was included to represent reduced functional reserve, mobility limitation, and self-management capacity. Functional deficits are important indicators of geriatric health burden and may indicate the need for broader clinical or community-health review. In this outcome, functional impairment complemented metabolic, renal–urinary, and medication-related indicators to capture screening-derived abnormality burden [1,6,10,11,33,36]. | This component is not a bowel-specific functional test and does not diagnose constipation, bowel dysfunction, frailty, or comprehensive geriatric impairment. It represents a limited routine-record functional indicator. |

**Note.** The components were selected to construct a screening-derived abnormality-burden outcome based on routinely available primary-care health-examination data. The component selection was informed by accumulation-of-deficits, multidimensional frailty, comprehensive geriatric assessment, multimorbidity, polypharmacy, metabolic-risk, renal-risk, and functional-limitation concepts [1,2,6,10,11,25,30,33–36]. The outcome was designed to capture clustered metabolic, renal–urinary, medication-related, and functional screening abnormalities in older women and to support primary-care risk recognition and follow-up prioritisation. It was not intended to diagnose constipation, estimate constipation prevalence, replace symptom-based bowel assessment, function as a validated frailty index, serve as a comprehensive geriatric assessment score, or represent a validated multidomain geriatric vulnerability phenotype. Each component should therefore be interpreted as a pragmatic screening abnormality rather than as a bowel-specific, disease-specific, or diagnostic criterion. Because this internally derived outcome has not been externally validated against symptom-defined constipation, formal frailty instruments, comprehensive geriatric assessment, healthcare utilisation, or prospective clinical events, its findings should be interpreted cautiously and in conjunction with the sensitivity analyses reported in the Supplementary Material.

**Supplementary Table S2. Female analytic sample, labour-type physical-activity distribution, and diabetes-related exposure across activity categories**

**Panel A. Overview of the female analytic sample and labour-type physical-activity distribution**

| **Item** | **Value** |
| --- | --- |
| Female analytic sample size | 1163 |
| High screening-derived abnormality burden, n (%) | 320 (27.52%) |
| Diabetes-related exposure, n (%) | 262 (22.53%) |
| Participants with available three-category labour-type PA classification, n | 1131 |
| Missing three-category labour-type PA classification, n (%) | 32 (2.75%) |
| Inactive, n | 432 |
| LowActive, n | 143 |
| HighActive, n | 556 |
| Inactive under binary PA robustness definition, n (%) | 432 (38.20%) |
| Median PA index among active participants | 180 |

**Panel B. Distribution of diabetes-related exposure across three-category labour-type physical-activity groups**

| **Labour-type physical-activity category** | **No diabetes-related exposure, n (%)** | **Diabetes-related exposure, n (%)** | **Total, n** |
| --- | --- | --- | --- |
| Inactive | 337 (78.01%) | 95 (21.99%) | 432 |
| LowActive | 111 (77.62%) | 32 (22.38%) | 143 |
| HighActive | 431 (77.52%) | 125 (22.48%) | 556 |

Note. PA, physical activity. The PA index was calculated as weekly activity frequency × session duration using routinely recorded health-examination information. Participants were classified as Inactive when PA index = 0. Among active participants, the median PA index was used to classify LowActive and HighActive groups. These categories represent relative labour-type physical-activity levels within this cohort and should not be interpreted as guideline-based physical-activity categories, validated exercise-dose levels, objectively measured activity levels, or measures of leisure-time health-promoting exercise. Percentages in Panel B were calculated within each labour-type physical-activity category. Participants with missing PA classification were excluded from Panel B. This table is descriptive and was used to characterise the analytic sample and the distribution of diabetes-related exposure across the cohort-specific labour-type PA categories.

**Supplementary Table S3. Prevalence of components included in the screening-derived abnormality-burden outcome**

| **Component indicator** | **Domain** | **Non-missing n** | **Positive n** | **Positive %** |
| --- | --- | --- | --- | --- |
| Elevated triglycerides | Metabolic burden | 1160 | 597 | 51.47 |
| Low HDL-C | Metabolic burden | 1157 | 499 | 43.13 |
| Abnormal BMI | Metabolic burden | 1161 | 183 | 15.76 |
| Renal abnormality | Renal–urinary burden | 1158 | 363 | 31.35 |
| Urinalysis occult blood positivity | Renal–urinary burden | 1163 | 421 | 36.20 |
| Polypharmacy | Medication-related burden | 1162 | 45 | 3.87 |
| Functional impairment | Functional limitation | 1163 | 40 | 3.44 |

Note. HDL-C, high-density lipoprotein cholesterol; BMI, body mass index. These seven indicators formed the screening-derived abnormality-burden outcome. Renal abnormality was operationalised as elevated creatinine and/or elevated urea, and urinalysis occult blood positivity was based on routine urinalysis coding. High screening-derived abnormality burden was defined as ≥3 abnormal components among participants with at least five non-missing component indicators. The table describes the component distribution of the abnormality-burden outcome and shows that it was mainly shaped by metabolic and renal–urinary abnormalities, whereas medication-related and functional components were less frequent. This internally derived outcome was not intended to diagnose constipation, estimate constipation prevalence, replace symptom-based bowel assessment, function as a validated frailty index, serve as a comprehensive geriatric assessment score, or represent a validated multidomain geriatric phenotype.

**Supplementary Table S4. Robustness analysis using binary labour-type physical-activity exposure**

| **Model** | **Adjustment strategy** | **Term** | **PR** | **95% CI** | **P value** | **N** |
| --- | --- | --- | --- | --- | --- | --- |
| Model 1: Crude | Unadjusted | Diabetes-related exposure | 1.751 | 1.448–2.118 | <0.001 | 1131 |
| Model 1: Crude | Unadjusted | Active vs Inactive | 1.095 | 0.900–1.332 | 0.365 | 1131 |
| Model 2: Age-adjusted | Age | Diabetes-related exposure | 1.740 | 1.436–2.107 | <0.001 | 1131 |
| Model 2: Age-adjusted | Age | Active vs Inactive | 1.063 | 0.871–1.298 | 0.547 | 1131 |
| Model 3: Primary adjusted without BMI | Age, hypertension, current smoking | Diabetes-related exposure | 1.669 | 1.369–2.036 | <0.001 | 1131 |
| Model 3: Primary adjusted without BMI | Age, hypertension, current smoking | Active vs Inactive | 1.043 | 0.853–1.275 | 0.681 | 1131 |
| Model 4: BMI-adjusted sensitivity | Age, hypertension, BMI, current smoking | Diabetes-related exposure | 1.616 | 1.331–1.962 | <0.001 | 1129 |
| Model 4: BMI-adjusted sensitivity | Age, hypertension, BMI, current smoking | Active vs Inactive | 1.022 | 0.840–1.243 | 0.828 | 1129 |

**Note.** PR, prevalence ratio; CI, confidence interval; BMI, body mass index. Modified Poisson regression with robust HC3 standard errors was used. The binary labour-type physical-activity exposure collapsed LowActive and HighActive into an Active category, with Inactive as the reference group. The primary analysis used the three-category labour-type physical-activity variable; this binary specification was used only as a robustness analysis of activity classification. Model 1 was unadjusted. Model 2 adjusted for age. Model 3 was the primary adjusted model and included age, hypertension, and current smoking. Continuous BMI was not included in Model 3 because abnormal BMI was one component of the screening-derived abnormality-burden outcome. Model 4 additionally adjusted for continuous BMI as a sensitivity analysis. The binary specification yielded a pattern consistent with the primary three-category analysis: diabetes-related exposure showed positive estimates across models, whereas binary labour-type physical activity was not independently associated with high abnormality burden.

**Supplementary Table S5. Sensitivity analyses using alternative thresholds for high screening-derived abnormality burden**

**Panel A. Prevalence of high abnormality burden under alternative thresholds**

| **Threshold** | **Non-missing n** | **Positive n** | **High abnormality burden %** |
| --- | --- | --- | --- |
| ≥2 abnormal components | 1163 | 717 | 61.65 |
| ≥3 abnormal components | 1163 | 320 | 27.52 |
| ≥4 abnormal components | 1163 | 86 | 7.39 |

**Panel B. Association models under alternative abnormality-burden thresholds**

| **Threshold** | **Model** | **Term** | **PR** | **95% CI** | **P value** | **N** |
| --- | --- | --- | --- | --- | --- | --- |
| ≥2 | Model 1: Crude | Diabetes-related exposure | 1.192 | 1.081–1.313 | <0.001 | 1131 |
| ≥2 | Model 1: Crude | LowActive vs Inactive | 0.984 | 0.846–1.144 | 0.831 | 1131 |
| ≥2 | Model 1: Crude | HighActive vs Inactive | 1.003 | 0.909–1.107 | 0.952 | 1131 |
| ≥2 | Model 2: Age-adjusted | Diabetes-related exposure | 1.189 | 1.079–1.311 | <0.001 | 1131 |
| ≥2 | Model 2: Age-adjusted | LowActive vs Inactive | 0.975 | 0.838–1.136 | 0.749 | 1131 |
| ≥2 | Model 2: Age-adjusted | HighActive vs Inactive | 0.993 | 0.897–1.100 | 0.899 | 1131 |
| ≥2 | Model 2: Age-adjusted | Age | 0.997 | 0.989–1.005 | 0.480 | 1131 |
| ≥2 | Model 3: BMI-adjusted sensitivity | Diabetes-related exposure | 1.147 | 1.038–1.269 | 0.007 | 1129 |
| ≥2 | Model 3: BMI-adjusted sensitivity | LowActive vs Inactive | 0.942 | 0.811–1.093 | 0.429 | 1129 |
| ≥2 | Model 3: BMI-adjusted sensitivity | HighActive vs Inactive | 0.968 | 0.876–1.070 | 0.526 | 1129 |
| ≥2 | Model 3: BMI-adjusted sensitivity | Age | 1.000 | 0.992–1.008 | 0.998 | 1129 |
| ≥2 | Model 3: BMI-adjusted sensitivity | Hypertension | 1.021 | 0.928–1.124 | 0.668 | 1129 |
| ≥2 | Model 3: BMI-adjusted sensitivity | BMI | 1.039 | 1.027–1.051 | <0.001 | 1129 |
| ≥2 | Model 3: BMI-adjusted sensitivity | Current smoking | 1.261 | 1.116–1.426 | <0.001 | 1129 |
| ≥3 | Model 1: Crude | Diabetes-related exposure | 1.751 | 1.448–2.119 | <0.001 | 1131 |
| ≥3 | Model 1: Crude | LowActive vs Inactive | 1.049 | 0.771–1.428 | 0.759 | 1131 |
| ≥3 | Model 1: Crude | HighActive vs Inactive | 1.106 | 0.902–1.357 | 0.331 | 1131 |
| ≥3 | Model 2: Age-adjusted | Diabetes-related exposure | 1.739 | 1.436–2.108 | <0.001 | 1131 |
| ≥3 | Model 2: Age-adjusted | LowActive vs Inactive | 1.024 | 0.749–1.399 | 0.883 | 1131 |
| ≥3 | Model 2: Age-adjusted | HighActive vs Inactive | 1.074 | 0.873–1.321 | 0.502 | 1131 |
| ≥3 | Model 2: Age-adjusted | Age | 0.992 | 0.975–1.009 | 0.347 | 1131 |
| ≥3 | Model 3: BMI-adjusted sensitivity | Diabetes-related exposure | 1.614 | 1.329–1.961 | <0.001 | 1129 |
| ≥3 | Model 3: BMI-adjusted sensitivity | LowActive vs Inactive | 0.966 | 0.716–1.302 | 0.819 | 1129 |
| ≥3 | Model 3: BMI-adjusted sensitivity | HighActive vs Inactive | 1.037 | 0.846–1.271 | 0.729 | 1129 |
| ≥3 | Model 3: BMI-adjusted sensitivity | Age | 0.996 | 0.979–1.013 | 0.638 | 1129 |
| ≥3 | Model 3: BMI-adjusted sensitivity | Hypertension | 1.058 | 0.870–1.287 | 0.572 | 1129 |
| ≥3 | Model 3: BMI-adjusted sensitivity | BMI | 1.084 | 1.058–1.111 | <0.001 | 1129 |
| ≥3 | Model 3: BMI-adjusted sensitivity | Current smoking | 1.209 | 0.890–1.643 | 0.224 | 1129 |
| ≥4 | Model 1: Crude | Diabetes-related exposure | 2.821 | 2.043–3.895 | <0.001 | 1131 |
| ≥4 | Model 1: Crude | LowActive vs Inactive | 1.250 | 0.687–2.276 | 0.465 | 1131 |
| ≥4 | Model 1: Crude | HighActive vs Inactive | 1.407 | 0.920–2.153 | 0.115 | 1131 |
| ≥4 | Model 2: Age-adjusted | Diabetes-related exposure | 2.787 | 2.016–3.854 | <0.001 | 1131 |
| ≥4 | Model 2: Age-adjusted | LowActive vs Inactive | 1.180 | 0.647–2.150 | 0.590 | 1131 |
| ≥4 | Model 2: Age-adjusted | HighActive vs Inactive | 1.313 | 0.850–2.030 | 0.218 | 1131 |
| ≥4 | Model 2: Age-adjusted | Age | 0.988 | 0.957–1.020 | 0.445 | 1131 |
| ≥4 | Model 3: BMI-adjusted sensitivity | Diabetes-related exposure | 2.392 | 1.720–3.328 | <0.001 | 1129 |
| ≥4 | Model 3: BMI-adjusted sensitivity | LowActive vs Inactive | 1.015 | 0.558–1.844 | 0.961 | 1129 |
| ≥4 | Model 3: BMI-adjusted sensitivity | HighActive vs Inactive | 1.194 | 0.777–1.834 | 0.420 | 1129 |
| ≥4 | Model 3: BMI-adjusted sensitivity | Age | 0.994 | 0.963–1.027 | 0.721 | 1129 |
| ≥4 | Model 3: BMI-adjusted sensitivity | Hypertension | 1.045 | 0.666–1.639 | 0.847 | 1129 |
| ≥4 | Model 3: BMI-adjusted sensitivity | BMI | 1.108 | 1.067–1.151 | <0.001 | 1129 |
| ≥4 | Model 3: BMI-adjusted sensitivity | Current smoking | 1.337 | 0.663–2.700 | 0.418 | 1129 |

**Note.** PR, prevalence ratio; CI, confidence interval; BMI, body mass index. Modified Poisson regression with robust HC3 standard errors was used. The primary definition of high screening-derived abnormality burden was ≥3 abnormal components. Alternative thresholds of ≥2 and ≥4 abnormal components were examined to assess threshold dependence of the primary binary outcome. Inactive was the reference category for labour-type physical activity. Model 1 was unadjusted. Model 2 adjusted for age. Model 3 was the BMI-adjusted sensitivity model and included age, hypertension, BMI, and current smoking. Diabetes-related exposure showed positive estimates across threshold definitions, whereas LowActive and HighActive did not show independent associations with high abnormality burden. These analyses were interpreted as threshold-dependence sensitivity analyses rather than as alternative primary outcome definitions.

**Supplementary Table S6. Sensitivity analyses addressing potential overlap between BMI as an outcome component and BMI as a covariate**

| **Analysis** | **Outcome definition** | **Adjustment strategy** | **Diabetes-related exposure PR** | **95% CI** | **P value** | **N** |
| --- | --- | --- | --- | --- | --- | --- |
| Primary adjusted model | High screening-derived abnormality burden, ≥3 components | Age, hypertension, current smoking; BMI not included | 1.668 | 1.367–2.035 | <0.001 | 1131 |
| BMI-adjusted sensitivity model | High screening-derived abnormality burden, ≥3 components | Age, hypertension, BMI, current smoking | 1.614 | 1.329–1.961 | <0.001 | 1129 |
| Excluding BMI-abnormality component | High abnormality burden recalculated after removing abnormal BMI from the abnormality-burden score | Age, hypertension, BMI, current smoking | 1.696 | 1.353–2.127 | <0.001 | 1129 |

**Note.** BMI, body mass index; PR, prevalence ratio; CI, confidence interval. Modified Poisson regression with robust HC3 standard errors was used. These analyses addressed potential overlap between BMI as an outcome component and BMI as a covariate. The primary adjusted model did not include continuous BMI because abnormal BMI was one of the components used to construct the screening-derived abnormality-burden outcome. The BMI-adjusted model was performed as a sensitivity analysis. An additional sensitivity analysis recalculated the abnormality-burden outcome after excluding the BMI-abnormality component and then included continuous BMI as a covariate. These analyses were interpreted as BMI-overlap sensitivity checks rather than as alternative primary outcome definitions. Across these specifications, diabetes-related exposure yielded directionally consistent positive estimates.

**Supplementary Table S7. Non-metabolic abnormality-burden analysis excluding metabolic components from the screening-derived abnormality-burden outcome**

**Panel A. Definition and prevalence of high non-metabolic abnormality burden**

| **Non-metabolic component** | **Domain** | **Included in non-metabolic abnormality-burden outcome** |
| --- | --- | --- |
| Renal abnormality | Renal–urinary burden | Yes |
| Urinalysis occult blood positivity | Renal–urinary burden | Yes |
| Polypharmacy | Medication-related burden | Yes |
| Functional impairment | Functional limitation | Yes |

| **Outcome definition** | **Non-missing n** | **Positive n** | **Positive %** |
| --- | --- | --- | --- |
| High non-metabolic abnormality burden, ≥2 abnormal non-metabolic components | 1163 | 168 | 14.45 |

**Panel B. Association models for high non-metabolic abnormality burden**

| **Model** | **Term** | **PR** | **95% CI** | **P value** | **N** |
| --- | --- | --- | --- | --- | --- |
| Model 1: Crude | Diabetes-related exposure | 1.557 | 1.149–2.110 | 0.004 | 1131 |
| Model 1: Crude | LowActive vs Inactive | 0.924 | 0.570–1.497 | 0.748 | 1131 |
| Model 1: Crude | HighActive vs Inactive | 1.013 | 0.746–1.374 | 0.936 | 1131 |
| Model 2: Age-adjusted | Diabetes-related exposure | 1.574 | 1.159–2.137 | 0.004 | 1131 |
| Model 2: Age-adjusted | LowActive vs Inactive | 0.957 | 0.588–1.558 | 0.861 | 1131 |
| Model 2: Age-adjusted | HighActive vs Inactive | 1.057 | 0.775–1.440 | 0.726 | 1131 |
| Model 2: Age-adjusted | Age | 1.013 | 0.990–1.037 | 0.268 | 1131 |
| Model 3: Primary adjusted without BMI | Diabetes-related exposure | 1.463 | 1.077–1.988 | 0.015 | 1131 |
| Model 3: Primary adjusted without BMI | LowActive vs Inactive | 0.918 | 0.562–1.501 | 0.733 | 1131 |
| Model 3: Primary adjusted without BMI | HighActive vs Inactive | 1.035 | 0.757–1.414 | 0.831 | 1131 |
| Model 3: Primary adjusted without BMI | Age | 1.013 | 0.989–1.036 | 0.293 | 1131 |
| Model 3: Primary adjusted without BMI | Hypertension | 1.303 | 0.965–1.758 | 0.084 | 1131 |
| Model 3: Primary adjusted without BMI | Current smoking | 0.779 | 0.433–1.401 | 0.404 | 1131 |
| Model 4: BMI-adjusted sensitivity | Diabetes-related exposure | 1.467 | 1.073–2.007 | 0.016 | 1129 |
| Model 4: BMI-adjusted sensitivity | LowActive vs Inactive | 0.931 | 0.569–1.523 | 0.775 | 1129 |
| Model 4: BMI-adjusted sensitivity | HighActive vs Inactive | 1.040 | 0.758–1.426 | 0.809 | 1129 |
| Model 4: BMI-adjusted sensitivity | Age | 1.011 | 0.987–1.035 | 0.388 | 1129 |
| Model 4: BMI-adjusted sensitivity | Hypertension | 1.314 | 0.970–1.781 | 0.078 | 1129 |
| Model 4: BMI-adjusted sensitivity | BMI | 0.978 | 0.930–1.028 | 0.372 | 1129 |
| Model 4: BMI-adjusted sensitivity | Current smoking | 0.785 | 0.437–1.410 | 0.418 | 1129 |

**Note.** PR, prevalence ratio; CI, confidence interval; BMI, body mass index. Modified Poisson regression with robust HC3 standard errors was used. The non-metabolic abnormality-burden outcome excluded the three metabolic components of the original screening-derived abnormality-burden outcome: elevated triglycerides, low HDL-C, and abnormal BMI. High non-metabolic abnormality burden was defined as ≥2 abnormalities among renal abnormality, urinalysis occult blood positivity, polypharmacy, and functional impairment. This analysis examined whether the diabetes-related association depended entirely on the metabolic components of the original abnormality-burden outcome. The association between diabetes-related exposure and high non-metabolic abnormality burden yielded directionally consistent positive estimates after adjustment, suggesting that the primary association was not fully explained by metabolic components alone. The non-metabolic abnormality-burden outcome remains exploratory and should not be interpreted as a validated clinical endpoint. Labour-type physical-activity categories were not independently associated with high non-metabolic abnormality burden.

**Supplementary Table S8. Domain-specific analyses of the screening-derived abnormality-burden outcome**

**Panel A. Definition and prevalence of domain-specific abnormality-burden outcomes**

| **Domain-specific abnormality-burden outcome** | **Components included** | **Operational definition** | **Non-missing n** | **Positive n** | **Positive %** |
| --- | --- | --- | --- | --- | --- |
| Metabolic burden | Elevated triglycerides, low HDL-C, abnormal BMI | ≥2 metabolic abnormalities | 1163 | 405 | 34.82 |
| Renal–urinary burden | Renal abnormality, urinalysis occult blood positivity | ≥1 renal–urinary abnormality | 1163 | 654 | 56.23 |
| Medication–functional burden | Polypharmacy, functional impairment | Polypharmacy and/or functional impairment | 1163 | 84 | 7.22 |

**Panel B. Association models for domain-specific abnormality-burden outcomes**

| **Domain-specific abnormality-burden outcome** | **Model** | **Term** | **PR** | **95% CI** | **P value** | **N** |
| --- | --- | --- | --- | --- | --- | --- |
| Metabolic burden | Primary adjusted without BMI | Diabetes-related exposure | 1.460 | 1.230–1.734 | <0.001 | 1131 |
| Metabolic burden | Primary adjusted without BMI | LowActive vs Inactive | 1.034 | 0.783–1.365 | 0.814 | 1131 |
| Metabolic burden | Primary adjusted without BMI | HighActive vs Inactive | 1.015 | 0.846–1.218 | 0.873 | 1131 |
| Renal–urinary burden | Primary adjusted without BMI | Diabetes-related exposure | 0.994 | 0.873–1.131 | 0.931 | 1131 |
| Renal–urinary burden | Primary adjusted without BMI | LowActive vs Inactive | 0.970 | 0.803–1.171 | 0.752 | 1131 |
| Renal–urinary burden | Primary adjusted without BMI | HighActive vs Inactive | 1.024 | 0.901–1.164 | 0.716 | 1131 |
| Medication–functional burden | Primary adjusted without BMI | Diabetes-related exposure | 4.794 | 3.178–7.232 | <0.001 | 1131 |
| Medication–functional burden | Primary adjusted without BMI | LowActive vs Inactive | 0.381 | 0.156–0.928 | 0.034 | 1131 |
| Medication–functional burden | Primary adjusted without BMI | HighActive vs Inactive | 0.551 | 0.337–0.902 | 0.018 | 1131 |

**Note.** PR, prevalence ratio; CI, confidence interval; BMI, body mass index. Modified Poisson regression with robust HC3 standard errors was used. Domain-specific abnormality-burden outcomes were constructed to examine whether the overall association pattern differed across component domains of the screening-derived abnormality-burden outcome. The primary adjusted models included diabetes-related exposure, three-category labour-type physical activity, age, hypertension, and current smoking, without continuous BMI adjustment. Metabolic burden was defined as ≥2 abnormalities among elevated triglycerides, low HDL-C, and abnormal BMI. Renal–urinary burden was defined as renal abnormality and/or urinalysis occult blood positivity. Medication–functional burden was defined as polypharmacy and/or functional impairment. Because the number of components and the prevalence of positive cases differed substantially across domains, especially for the relatively infrequent medication–functional burden outcome, these analyses should be interpreted only as exploratory evidence of component contribution and outcome consistency. They should not be used to infer domain-specific mechanisms, causal pathways, or a protective effect of labour-type physical activity on medication–functional burden. These analyses were intended to support cautious interpretation of the internally derived abnormality-burden outcome rather than to redefine the primary outcome.

**Supplementary Table S9. Leave-one-component-out sensitivity analyses for the screening-derived abnormality-burden outcome**

| **Excluded component** | **Components retained in recalculated abnormality-burden outcome** | **Diabetes-related exposure PR** | **95% CI** | **P value** | **N** |
| --- | --- | --- | --- | --- | --- |
| Elevated triglycerides | Low HDL-C, abnormal BMI, renal abnormality, urinalysis occult blood positivity, polypharmacy, functional impairment | 1.954 | 1.424–2.682 | <0.001 | 1131 |
| Low HDL-C | Elevated triglycerides, abnormal BMI, renal abnormality, urinalysis occult blood positivity, polypharmacy, functional impairment | 1.953 | 1.423–2.682 | <0.001 | 1131 |
| Abnormal BMI | Elevated triglycerides, low HDL-C, renal abnormality, urinalysis occult blood positivity, polypharmacy, functional impairment | 1.696 | 1.353–2.127 | <0.001 | 1129 |
| Renal abnormality | Elevated triglycerides, low HDL-C, abnormal BMI, urinalysis occult blood positivity, polypharmacy, functional impairment | 1.951 | 1.524–2.497 | <0.001 | 1131 |
| Urinalysis occult blood positivity | Elevated triglycerides, low HDL-C, abnormal BMI, renal abnormality, polypharmacy, functional impairment | 2.258 | 1.743–2.925 | <0.001 | 1129 |
| Polypharmacy | Elevated triglycerides, low HDL-C, abnormal BMI, renal abnormality, urinalysis occult blood positivity, functional impairment | 1.497 | 1.226–1.829 | <0.001 | 1131 |
| Functional impairment | Elevated triglycerides, low HDL-C, abnormal BMI, renal abnormality, urinalysis occult blood positivity, polypharmacy | 1.676 | 1.373–2.044 | <0.001 | 1131 |

**Note.** PR, prevalence ratio; CI, confidence interval; BMI, body mass index; HDL-C, high-density lipoprotein cholesterol. Modified Poisson regression with robust HC3 standard errors was used. Each row represents a sensitivity analysis in which one component was removed from the screening-derived abnormality-burden outcome and high abnormality burden was recalculated using the remaining components. The models were adjusted for age, hypertension, and current smoking. The analysis excluding the BMI-abnormality component additionally allowed assessment of potential overlap between BMI as an outcome component and BMI as a covariate, with continuous BMI adjustment where applicable. These analyses evaluated whether the diabetes-related association was eliminated by removal of any single component. The findings should be interpreted as component-dependence robustness checks rather than as validation of the internally derived abnormality-burden outcome as a clinical endpoint, bowel-specific outcome, or validated frailty measure.

Supplementary Table S10. Sensitivity analyses using alternative diabetes-related exposure definitions

**Panel A. Alternative diabetes-related exposure definitions**

| **Exposure definition** | **Operational definition** | **Purpose of sensitivity analysis** |
| --- | --- | --- |
| Primary diabetes-related exposure | Self-reported diabetes, fasting plasma glucose ≥7.0 mmol/L, or current use of glucose-lowering medication | Primary screening-based definition of diabetes-related glycaemic burden |
| Diabetes-related exposure without medication use | Self-reported diabetes or fasting plasma glucose ≥7.0 mmol/L | To assess whether the association depended on glucose-lowering medication use |
| Fasting plasma glucose-defined exposure only | Fasting plasma glucose ≥7.0 mmol/L | To assess whether the association was present using laboratory-defined glycaemic burden only |
| Self-reported diabetes only | Self-reported history of diabetes | To assess whether the association was present using history-based diabetes information only |

**Panel B. Association between alternative diabetes-related exposure definitions and high screening-derived abnormality burden**

| **Exposure definition** | **Adjustment strategy** | **PR** | **95% CI** | **P value** | **N** |
| --- | --- | --- | --- | --- | --- |
| Primary diabetes-related exposure | Age, hypertension, current smoking | 1.668 | 1.367–2.035 | <0.001 | 1131 |
| Diabetes-related exposure without medication use | Age, hypertension, current smoking | 1.686 | 1.382–2.058 | <0.001 | 1131 |
| Fasting plasma glucose-defined exposure only | Age, hypertension, current smoking | 1.650 | 1.339–2.033 | <0.001 | 1131 |
| Self-reported diabetes only | Age, hypertension, current smoking | 1.746 | 1.397–2.181 | <0.001 | 1131 |

Note. PR, prevalence ratio; CI, confidence interval. Modified Poisson regression with robust HC3 standard errors was used. The primary adjusted model included the exposure definition of interest, three-category labour-type physical activity, age, hypertension, and current smoking. Continuous BMI was not included in the primary adjusted model because abnormal BMI was one component of the screening-derived abnormality-burden outcome. These analyses evaluated whether the association between diabetes-related exposure and high abnormality burden depended on glucose-lowering medication use or on a specific screening-based diabetes-exposure operational definition. These definitions captured diabetes-related exposure or glycaemic screening burden in routine health-examination data and did not distinguish diabetes duration, severity, glycaemic history, treatment intensity, or adjudicated complication status. Across alternative definitions, diabetes-related exposure yielded directionally consistent positive estimates.

**Supplementary Table S11. Missing-data profile, complete-case comparison, and multiple-imputation sensitivity analysis**

**Panel A. Missing-data profile for variables used in primary and supplementary analyses**

| **Variable** | **Missing n** | **Missing %** |
| --- | --- | --- |
| High screening-derived abnormality burden | 0 | 0.00 |
| Diabetes-related exposure | 0 | 0.00 |
| Three-category labour-type PA classification | 32 | 2.75 |
| Binary labour-type PA classification | 32 | 2.75 |
| Age | 0 | 0.00 |
| Hypertension | 0 | 0.00 |
| BMI | 2 | 0.17 |
| Current smoking | 0 | 0.00 |
| Elevated triglycerides | 3 | 0.26 |
| Low HDL-C | 6 | 0.52 |
| Abnormal BMI | 2 | 0.17 |
| Renal abnormality | 5 | 0.43 |
| Urinalysis occult blood positivity | 0 | 0.00 |
| Polypharmacy | 1 | 0.09 |
| Functional impairment | 0 | 0.00 |

**Panel B. Comparison of participants included in and excluded from complete-case regression models**

| **Complete-case definition** | **Variable** | **Included** | **Excluded** |
| --- | --- | --- | --- |
| Primary adjusted model without BMI | N | 1131 | 32 |
| Primary adjusted model without BMI | Age, years | 72.56 ± 5.88 | 72.19 ± 5.84 |
| Primary adjusted model without BMI | High abnormality burden, n (%) | 311 (27.50%) | 9 (28.12%) |
| Primary adjusted model without BMI | Diabetes-related exposure, n (%) | 252 (22.28%) | 10 (31.25%) |
| Primary adjusted model without BMI | BMI, kg/m² | 24.50 ± 3.38 | 24.66 ± 3.22 |
| Primary adjusted model without BMI | Hypertension, n (%) | 580 (51.28%) | 5 (15.62%) |
| Primary adjusted model without BMI | Current smoking, n (%) | 87 (7.69%) | 5 (15.62%) |
| BMI-adjusted sensitivity model | N | 1129 | 34 |
| BMI-adjusted sensitivity model | Age, years | 72.56 ± 5.88 | 71.97 ± 5.74 |
| BMI-adjusted sensitivity model | High abnormality burden, n (%) | 310 (27.46%) | 10 (29.41%) |
| BMI-adjusted sensitivity model | Diabetes-related exposure, n (%) | 250 (22.14%) | 12 (35.29%) |
| BMI-adjusted sensitivity model | BMI, kg/m² | 24.50 ± 3.38 | 24.66 ± 3.22 |
| BMI-adjusted sensitivity model | Hypertension, n (%) | 579 (51.28%) | 6 (17.65%) |
| BMI-adjusted sensitivity model | Current smoking, n (%) | 87 (7.71%) | 5 (14.71%) |

**Panel C. Multiple-imputation sensitivity analysis**

| **Model** | **Term** | **PR** | **95% CI** | **P value** | **N** |
| --- | --- | --- | --- | --- | --- |
| MICE pooled BMI-adjusted model | Diabetes-related exposure | 1.577 | 1.301–1.912 | <0.001 | 1163 |
| MICE pooled BMI-adjusted model | LowActive vs Inactive | 0.954 | 0.706–1.290 | 0.761 | 1163 |
| MICE pooled BMI-adjusted model | HighActive vs Inactive | 1.036 | 0.845–1.269 | 0.733 | 1163 |
| MICE pooled BMI-adjusted model | Age | 0.998 | 0.981–1.015 | 0.788 | 1163 |
| MICE pooled BMI-adjusted model | Hypertension | 1.060 | 0.875–1.284 | 0.549 | 1163 |
| MICE pooled BMI-adjusted model | BMI | 1.084 | 1.058–1.110 | <0.001 | 1163 |
| MICE pooled BMI-adjusted model | Current smoking | 1.228 | 0.913–1.651 | 0.174 | 1163 |

**Note.** PA, physical activity; BMI, body mass index; HDL-C, high-density lipoprotein cholesterol; MICE, multiple imputation by chained equations; PR, prevalence ratio; CI, confidence interval. Panel A summarises missingness for primary exposures, outcome components, and model covariates. Panel B compares participants included in and excluded from the primary complete-case model and the BMI-adjusted sensitivity model. Because the number of excluded participants was small, these comparisons were used descriptively rather than as formal missingness-mechanism tests. Panel C presents the multiple-imputation sensitivity analysis. Modified Poisson regression with robust HC3 standard errors was used, and estimates from imputed datasets were pooled using Rubin’s rules. Missingness was low overall, with the highest missing rate observed for labour-type PA classification. Although some differences were observed between included and excluded participants, the multiple-imputation analysis yielded estimates directionally consistent with the complete-case analyses.

**Supplementary Table S12. Ordinal and count-burden sensitivity analyses of the screening-derived abnormality-burden outcome**

**Panel A. Ordinal burden model**

| **Term** | **OR** | **95% CI** | **P value** | **N** |
| --- | --- | --- | --- | --- |
| Diabetes-related exposure | 1.835 | 1.397–2.410 | <0.001 | 1131 |
| LowActive vs Inactive | 0.935 | 0.653–1.337 | 0.712 | 1131 |
| HighActive vs Inactive | 1.002 | 0.786–1.277 | 0.987 | 1131 |
| Age | 0.990 | 0.971–1.009 | 0.295 | 1131 |
| Hypertension | 1.182 | 0.944–1.479 | 0.145 | 1131 |
| Current smoking | 1.618 | 1.089–2.402 | 0.017 | 1131 |

**Panel B. Count-burden Poisson model**

| **Term** | **RR** | **95% CI** | **P value** | **N** |
| --- | --- | --- | --- | --- |
| Diabetes-related exposure | 1.244 | 1.144–1.353 | <0.001 | 1131 |
| LowActive vs Inactive | 0.998 | 0.884–1.127 | 0.975 | 1131 |
| HighActive vs Inactive | 1.017 | 0.939–1.102 | 0.674 | 1131 |
| Age | 0.999 | 0.993–1.005 | 0.751 | 1131 |
| Hypertension | 1.069 | 0.991–1.153 | 0.082 | 1131 |
| Current smoking | 1.150 | 1.025–1.289 | 0.017 | 1131 |

**Note.** OR, odds ratio; RR, rate ratio; CI, confidence interval. The ordinal burden model treated the screening-derived abnormality-burden outcome as an ordered categorical outcome rather than as a binary high-abnormality-burden outcome. The count-burden Poisson model treated the abnormality-burden score as a count-based burden measure. Both models included diabetes-related exposure, three-category labour-type physical activity, age, hypertension, and current smoking. Continuous BMI was not included because abnormal BMI was one component of the abnormality-burden outcome. These analyses evaluated whether the main findings depended on the primary binary definition of high abnormality burden. Diabetes-related exposure showed positive estimates in both ordinal and count-burden analyses, whereas labour-type physical-activity categories were not independently associated with abnormality burden. These burden-scale analyses were interpreted as sensitivity analyses and were not used to redefine the primary outcome.

**Supplementary Table S13. Additional covariate-adjusted sensitivity analysis**

| **Model** | **Term** | **PR** | **95% CI** | **P value** | **N** |
| --- | --- | --- | --- | --- | --- |
| Additional covariate-adjusted model | Diabetes-related exposure | 1.639 | 1.349–1.992 | <0.001 | 1129 |
| Additional covariate-adjusted model | LowActive vs Inactive | 0.966 | 0.716–1.303 | 0.820 | 1129 |
| Additional covariate-adjusted model | HighActive vs Inactive | 1.031 | 0.841–1.264 | 0.771 | 1129 |
| Additional covariate-adjusted model | Age | 0.997 | 0.980–1.015 | 0.766 | 1129 |
| Additional covariate-adjusted model | Hypertension | 1.048 | 0.863–1.273 | 0.635 | 1129 |
| Additional covariate-adjusted model | BMI | 1.084 | 1.058–1.111 | <0.001 | 1129 |
| Additional covariate-adjusted model | Current smoking | 1.202 | 0.884–1.634 | 0.241 | 1129 |
| Additional covariate-adjusted model | Systolic blood pressure | 0.998 | 0.994–1.003 | 0.487 | 1129 |
| Additional covariate-adjusted model | Diastolic blood pressure | 1.003 | 0.994–1.012 | 0.487 | 1129 |
| Additional covariate-adjusted model | Ultrasound abnormality | 1.114 | 0.923–1.345 | 0.259 | 1129 |
| Additional covariate-adjusted model | Cognitive impairment | 1.097 | 0.796–1.513 | 0.572 | 1129 |

**Note.** PR, prevalence ratio; CI, confidence interval; BMI, body mass index. Modified Poisson regression with robust HC3 standard errors was used. This additional covariate-adjusted sensitivity model further adjusted for systolic blood pressure, diastolic blood pressure, routine ultrasound abnormality, and cognitive impairment, in addition to diabetes-related exposure, three-category labour-type physical activity, age, hypertension, BMI, and current smoking. This analysis examined whether the diabetes-related association with high screening-derived abnormality burden was materially altered after inclusion of additional routinely available screening variables. Diabetes-related exposure yielded a directionally consistent positive estimate, whereas labour-type physical-activity categories remained non-significant. Because some potentially important confounders, including diet quality, hydration, depressive symptoms, bowel symptoms, objectively measured physical activity, occupational workload, leisure-time exercise, and detailed medication classes, were unavailable, residual confounding cannot be excluded.

**Supplementary Table S14. Exploratory leakage-aware internal prioritisation analysis**

**Panel A. Leakage-aware predictor set used in the supplementary internal prioritisation analysis**

| **Predictor** | **Predictor role** | **Included in abnormality-burden outcome definition** | **Rationale for inclusion** |
| --- | --- | --- | --- |
| Age | Background variable | No | Routinely available demographic variable |
| Systolic blood pressure | Background screening variable | No | Routinely available cardiovascular screening indicator |
| Diastolic blood pressure | Background screening variable | No | Routinely available cardiovascular screening indicator |
| Heart rate | Background screening variable | No | Routinely available physiological screening indicator |
| Hypertension | Background comorbidity variable | No | Routinely recorded chronic disease marker |
| Diabetes-related exposure | Primary exposure variable | No | Screening-based glycaemic burden indicator |
| Three-category labour-type physical activity | Primary exposure variable | No | Cohort-specific labour-type activity indicator |
| Current smoking | Background lifestyle variable | No | Routinely recorded lifestyle factor |
| ECG abnormality | Background screening variable | No | Routinely available screening abnormality |
| Ultrasound abnormality | Background screening variable | No | Routinely available screening abnormality |
| Cognitive impairment | Background functional/cognitive variable | No | Routinely available cognitive screening indicator |

**Panel B. Internal prioritisation performance of leakage-aware models**

| **Model** | **AUC** | **PR-AUC** | **Brier score** | **Calibration intercept** | **Calibration slope** |
| --- | --- | --- | --- | --- | --- |
| Ridge logistic regression | 0.595 | 0.384 | 0.205 | 0.154 | 1.014 |
| Logistic regression | 0.592 | 0.385 | 0.205 | -0.337 | 0.575 |
| Random forest | 0.538 | 0.357 | 0.220 | -0.706 | 0.199 |
| XGBoost | 0.527 | 0.358 | 0.221 | -0.771 | 0.142 |

**Panel C. Bootstrap optimism-corrected AUC for the logistic model**

| **Apparent AUC** | **Mean optimism** | **Optimism-corrected AUC** | **Bootstrap resamples** |
| --- | --- | --- | --- |
| 0.612 | 0.025 | 0.587 | 200 |

**Panel D. Threshold-based performance of the logistic model**

| **Threshold** | **Sensitivity** | **Specificity** | **PPV** | **NPV** | **Accuracy** | **F1 score** |
| --- | --- | --- | --- | --- | --- | --- |
| 0.10 | 1.000 | 0.000 | 0.276 | NA | 0.276 | 0.433 |
| 0.15 | 0.938 | 0.069 | 0.278 | 0.756 | 0.309 | 0.429 |
| 0.20 | 0.812 | 0.221 | 0.285 | 0.756 | 0.384 | 0.422 |
| 0.25 | 0.573 | 0.500 | 0.305 | 0.754 | 0.520 | 0.398 |
| 0.30 | 0.344 | 0.744 | 0.339 | 0.748 | 0.633 | 0.342 |
| 0.40 | 0.042 | 0.974 | 0.382 | 0.729 | 0.716 | 0.075 |
| 0.50 | 0.000 | 1.000 | NA | 0.724 | 0.724 | NA |

**Note.** AUC, area under the receiver operating characteristic curve; PR-AUC, precision–recall area under the curve; PPV, positive predictive value; NPV, negative predictive value; F1, F1 score; ECG, electrocardiogram. This supplementary analysis used a leakage-aware predictor set that excluded variables directly used to define the screening-derived abnormality-burden outcome, including triglycerides, HDL-C, BMI abnormality, renal abnormality, urinalysis occult blood positivity, polypharmacy, and functional impairment. For held-out testing, imputation parameters were estimated within the training data and then applied to the test data to reduce test-set information leakage. These models were intended only for exploratory internal prioritisation and transparency. They were not designed as diagnostic tools, externally validated prediction models, prognostic models, or deployment-ready screening algorithms. NA indicates that the metric was not estimable because no participants were classified in the relevant prediction category at that threshold. Overall discrimination was modest, and these results were not used to support the primary inference.

**Supplementary Table S15. Joint exposure pattern of diabetes-related exposure and binary labour-type physical activity in relation to high screening-derived abnormality burden**

| **Joint exposure group** | **n** | **High abnormality burden n** | **High abnormality burden %** | **95% CI for prevalence** |
| --- | --- | --- | --- | --- |
| Active + no diabetes-related exposure | 542 | 133 | 24.54 | 21.10–28.33 |
| Inactive + no diabetes-related exposure | 337 | 74 | 21.96 | 17.87–26.68 |
| Active + diabetes-related exposure | 157 | 66 | 42.04 | 34.60–49.86 |
| Inactive + diabetes-related exposure | 95 | 38 | 40.00 | 30.72–50.05 |

**Note.** CI, confidence interval. Joint exposure groups were constructed using the binary labour-type PA robustness definition and diabetes-related exposure status. Percentages indicate the prevalence of high screening-derived abnormality burden within each joint exposure stratum. The 95% CIs were calculated for descriptive prevalence estimates. Only participants with complete joint exposure information were included. This analysis was descriptive and was used to show the distribution of high screening-derived abnormality burden across combined diabetes-related exposure and binary labour-type PA strata. It was not intended to establish causal interaction.
