# Supplementary material for: Diabetes-related exposure and screening-derived abnormality burden among older rural women in Northeast China: a secondary analysis with contextual labour-type physical activity assessment
Source: Front Public Health. 2026 Jul 1;14:1841622. doi: 10.3389/fpubh.2026.1841622 (PMC13371314; doi:10.3389/fpubh.2026.1841622)
Supplement: Supplementary file 2 [file Table_2.DOCX]

**
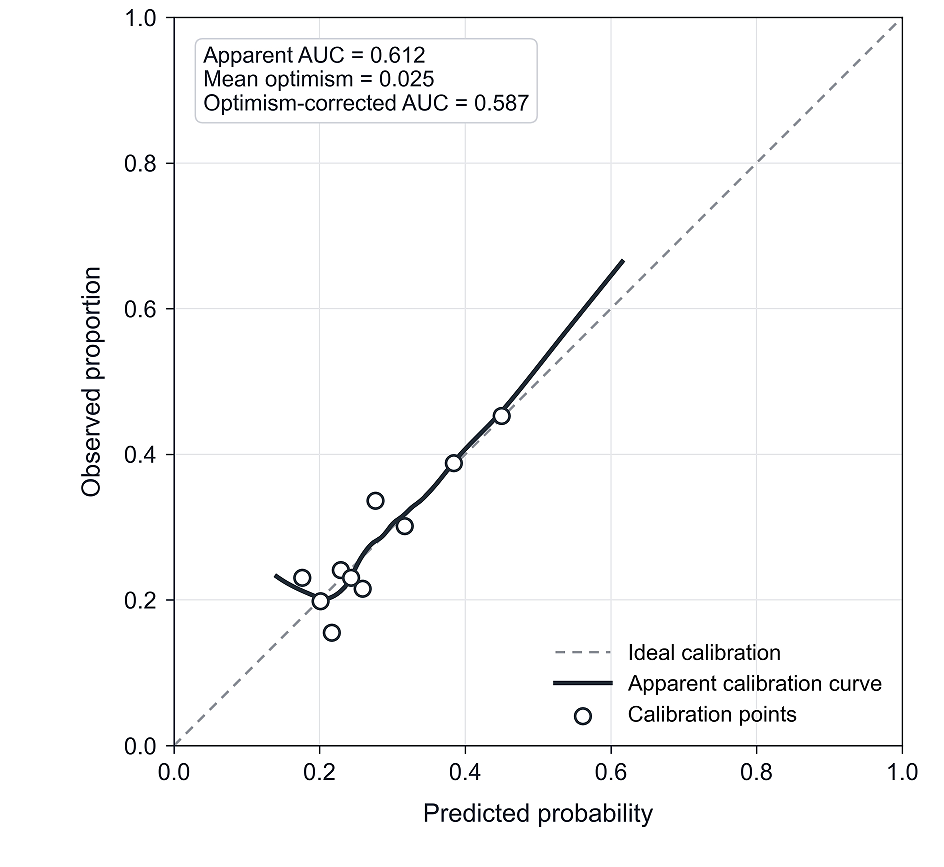
**

**Supplementary Figure S1. Calibration plot for the leakage-free logistic model.** Calibration plot for the leakage-free logistic model in the supplementary exploratory internal prediction analysis of high screening-derived abnormality burden. The dashed 45° line indicates ideal calibration. The apparent calibration curve shows the agreement between predicted probabilities and observed proportions. Predictor variables directly used to define the screening-derived abnormality-burden outcome were excluded from the model. Bootstrap internal validation yielded an apparent AUC of 0.612, mean optimism of 0.025, and optimism-corrected AUC of 0.587. This figure is intended to describe internal calibration only and does not support diagnostic or deployment-ready prediction.


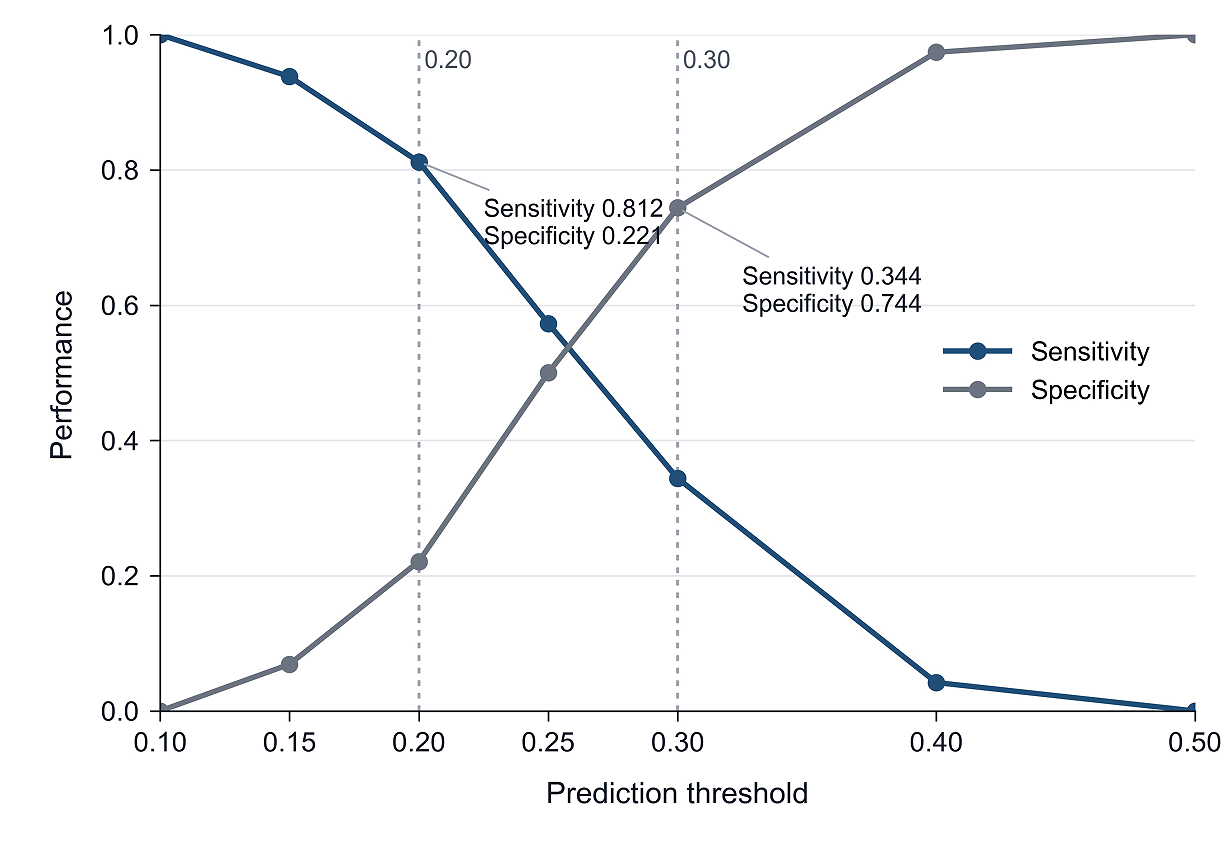


**Supplementary Figure S2. Threshold-based sensitivity and specificity of the leakage-free logistic model.** Threshold-based sensitivity and specificity of the leakage-free logistic model in the supplementary exploratory internal prediction analysis of high screening-derived abnormality burden. The curves illustrate the trade-off between sensitivity and specificity across candidate probability thresholds. At a threshold of 0.20, sensitivity was 0.812 and specificity was 0.221; at a threshold of 0.30, sensitivity was 0.344 and specificity was 0.744. These thresholds were examined for descriptive internal prioritisation only and were not intended to define clinical decision thresholds.


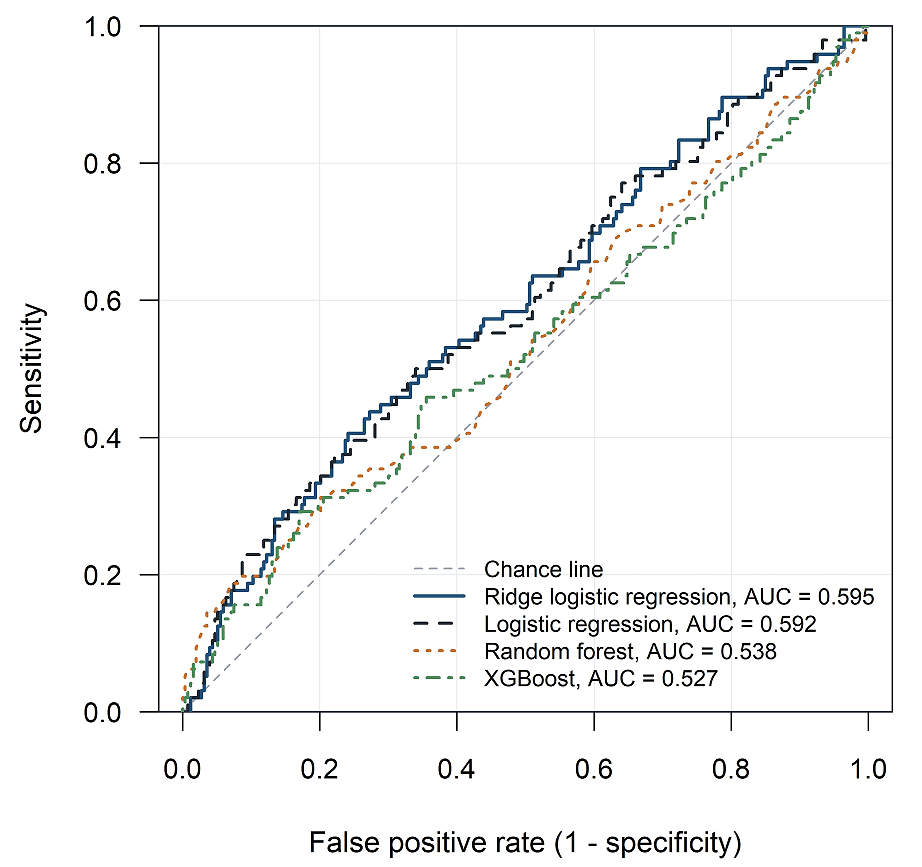


**Supplementary Figure S3. Receiver operating characteristic curves of leakage-free internal prediction models.** Receiver operating characteristic curves of four leakage-free internal prediction models in the supplementary exploratory analysis of high screening-derived abnormality burden. Predictor sets excluded variables directly used to define the screening-derived abnormality-burden outcome. Ridge logistic regression and logistic regression showed the highest discrimination, with AUC values of 0.595 and 0.592, respectively, whereas random forest and XGBoost showed lower discrimination, with AUC values of 0.538 and 0.527. Overall discrimination was modest across models, supporting interpretation of these analyses as exploratory internal prioritisation rather than diagnostic or deployment-ready prediction.


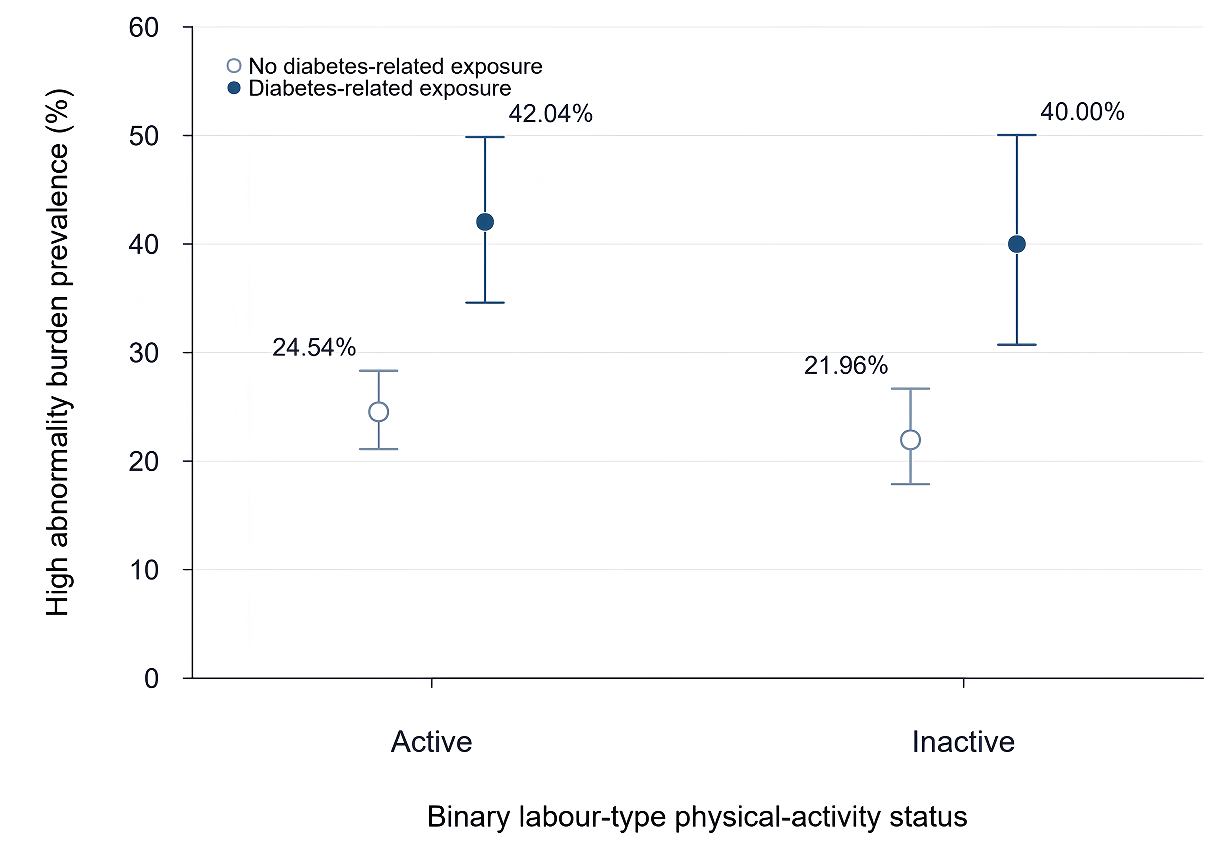


**Supplementary Figure S4. Descriptive prevalence of high screening-derived abnormality burden according to diabetes-related exposure and binary labour-type physical activity.** Dot-and-error plot showing the prevalence of high screening-derived abnormality burden and corresponding 95% confidence intervals within each descriptive stratum. Strata were defined using diabetes-related exposure and the binary labour-type physical-activity classification. This figure was descriptive only and was not interpreted as evidence of causal interaction, a primary physical-activity effect, or a repetition of previous labour-focused analyses.
